# Pharmacological effects and mechanisms of bee venom and its main components: Recent progress and perspective

**DOI:** 10.3389/fphar.2022.1001553

**Published:** 2022-09-27

**Authors:** Peiying Shi, Shihui Xie, Jiali Yang, Yi Zhang, Shuo Han, Songkun Su, Hong Yao

**Affiliations:** ^1^ Department of Traditional Chinese Medicine Resource and Bee Products, College of Animal Sciences (College of Bee Science), Fujian Agriculture and Forestry University, Fuzhou, China; ^2^ State and Local Joint Engineering Laboratory of Natural Biotoxins, Fujian Agriculture and Forestry University, Fuzhou, China; ^3^ Department of Pharmaceutical Analysis, School of Pharmacy, Fujian Medical University, Fuzhou, China

**Keywords:** bee venom, melittin, phospholipase A_2_, pharmacological effect, effect mechanism, bioinformatic analysis

## Abstract

Bee venom (BV), a type of defensive venom, has been confirmed to have favorable activities, such as anti-tumor, neuroprotective, anti-inflammatory, analgesic, anti-infectivity effects, etc. This study reviewed the recent progress on the pharmacological effects and mechanisms of BV and its main components against cancer, neurological disorders, inflammatory diseases, pain, microbial diseases, liver, kidney, lung and muscle injury, and other diseases in literature during the years 2018–2021. The related target proteins of BV and its main components against the diseases include Akt, mTOR, JNK, Wnt-5α, HIF-1α, NF-κB, JAK2, Nrf2, BDNF, Smad2/3, AMPK, and so on, which are referring to PI3K/Akt/mTOR, MAPK, Wnt/β-catenin, HIF-1α, NF-κB, JAK/STAT, Nrf2/HO-1, TrkB/CREB/BDNF, TGF-β/Smad2/3, and AMPK signaling pathways, etc. Further, with the reported targets, the potential effects and mechanisms on diseases were bioinformatically predicted via Kyoto Encyclopedia of Genes and Genomes (KEGG) pathway, disease ontology semantic and enrichment (DOSE) and protein-protein interaction (PPI) analyses. This review provides new insights into the therapeutic effects and mechanisms of BV and its main components on diseases.

## Introduction

Under the dual selection of natural selection and genetic evolution, the animal kingdom has long evolved a unique defensive venom system (Casewell et al., 2013). Bee venom (BV), a type of defensive venom, is generated in the bee’s venom glands and stored in the abdominal poison sac. (e.g., *Apis mellifera*) (Aufschnaiter et al., 2020). BV is a clear liquid with bitter taste, strong fragrance, pH value at 4.5–5.5, and specific gravity of 1.13, which is prone to volatilize and crystallize in the air (Khalil et al., 2021). BV contains smaller proteins, peptides and enzymes such as melittin (MEL), apamin, phospholipase A_2_ (PLA_2_) and other components referring to amines, sugars and minerals (Aufschnaiter et al., 2020; Khalil et al., 2021).

Based on these active components, BV has multiple diverse pharmacological effects. Some reviews have retrieved the pharmacological progress on one or a few aspects of BV, mainly referring to anti-tumor (Dutta et al., 2019; Mirzaei et al., 2021), neuroprotective (El-Seedi H. R. et al., 2020), anti-inflammatory (Dutta et al., 2019), analgesic (Kim and Han, 2020), anti-infectivity effects (El-Seedi H. et al., 2020), improving wound healing (Kurek-Gorecka et al., 2021), and other effects. Recently, Khalil et al. (2021) also summarized the therapeutic effects of BV in treatment of cancers, multiple sclerosis, dementia, osteoarthritis, rheumatoid arthritis (RA), and wounds, etc. These demonstrates that BV has a wide range of clinical applications could be attributed to its multi-target and multi-pathway characteristics. However, so far, there is still a lack of comprehensive and systematic pharmacological analysis of BV with multiple targets and pathways, which is unbeneficial to understanding the integrative pharmacological effect and mechanism of BV and its main components on diseases.

In the past 10 years, bioinformatic analyses, e.g., Encyclopedia of Genes and Genomes (KEGG) pathway, disease ontology semantic and enrichment (DOSE) and protein-protein interaction (PPI) analyses, etc., have been widely used in the investigation fields of genomics and proteomics, due to that they can comprehensively discover the biological mysteries of large and complex biological data accounting for physiological and pathological alternations of organism, or changes of organism in response to external stimuli (Yu et al., 2015; Wen et al., 2022). For the bioinformatic analyses, differentially expressed miRNAs (differentially expressed genes (DEGs) or differentially expressed proteins (DEPs) from omics experiments are screened firstly, and KEGG and disease ontology (DO) databases can then be called online by R language platform with the screened DEGs or DEPs to identify enriched pathways and related diseases usually using a two-tailed Fisher’s exact test. Meanwhile, all DEGs or DEPs can be searched against the STRING database for protein-protein interactions and can be visualized in R package to predict the key hub targets (genes or proteins). At present, by means of these bioinformatic ideas and tools, the potential therapeutic effects and mechanisms of several natural active ingredients, such as ginsenoside Rb_1_, Re and Tanshinone IIA have been analyzed systematically and deeply through mining their reported targets and pathways from literature, which indeed provide a lot of inspiration and clues for the future study of these ingredients (Zhong et al., 2021; Cai et al., 2022; Lin et al., 2022). Reasonably, with the help of bioinformatic tools, it should also be able to comprehensively understand the therapeutic effects and potential targets and mechanisms of the main ingredients in BV by mining their reported targets and pathways from previous reports.

Hence, in this paper, articles published from 2018 to 2021 and archived in Web of Science and PubMed databases were searched mainly using the keywords “bee venom and pharmacology,” supplemented with the keywords “bee venom and cancer” and “melittin and cancer,” and the duplicate articles were excluded. Based on these articles, we reviewed the current progress mainly from year 2018–2021 on the investigation of pharmacological effects and mechanisms of BV and its main components, mainly MEL, bvPLA_2_ and apamin. The reported action targets and pathways of them against cancer, neurological disorders, inflammatory diseases, pain, microbial diseases, liver, kidney, lung and muscle injury, and other diseases were summarized. Further, the possible anti-ill mechanisms of BV and its main components were comprehensively and systematically studied through DOSE, KEGG pathway, and PPI analyses according to the reported targets. The present study has deeply understood the pharmacological effects and mechanisms of BV and its main components against ills, which will help to promote the development and clinical application for BV.

## Main components of bee venom

**FIGURE 1:**
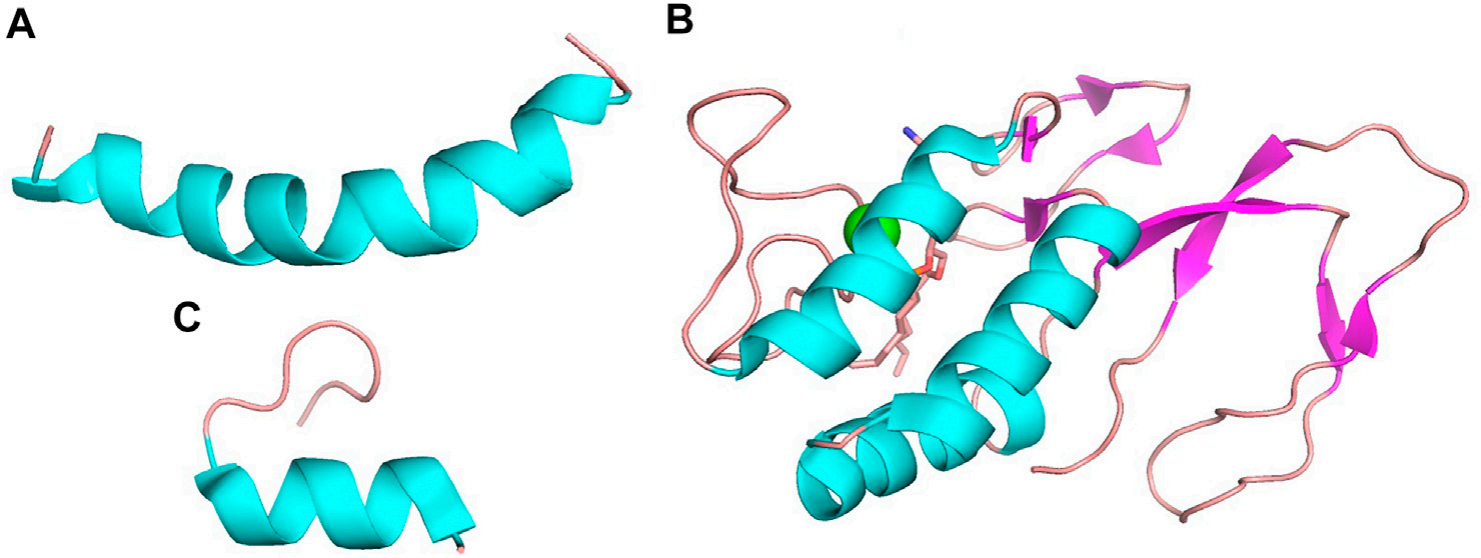
The structures of **(A)** MEL (PDB ID 6dst), **(B)** bvPLA_2_ (PDB ID 1poc) and **(C)** apamin (PDB ID 7oxf). Structure **(A)** and **(C)** appear to be dominated by α-helix while structure **(B)** is dominated by a combination of α-helix and β-pleated sheet.

MEL, bvPLA_2_ and apamin are three main components in BV, which are the important material basis for BV to exert its pharmacological effects, and their structures are shown in Figure 1.

### Melittin

MEL is the dominant component, which is consisting of 26-residue peptide and representing about 40%–60% BV’s dry weight (Wehbe et al., 2019). The carboxyl terminal of MEL contains positively charged amino acids, while the amino terminal is hydrophobic. Therefore, it contains both hydrophilic and hydrophobic properties. Both the MEL molecules and the membrane-bound MEL are through α spirally connected (Raghuraman and Chattopadhyay, 2007). Apart from its non-specific biofilm dissolution characteristics (Carpena et al., 2020), it has significant antibacterial, anti-tumor, and other effects (Pashaei et al., 2019; Yu et al., 2020).

### Bee venom phospholipase A_2_

PLA_2_ is a 128 amino acids single polypeptide chain containing four disulfide bridges. The bvPLA_2_ pertains to the group III secretory PLA_2_ (sPLA_2_) enzymes, accounting for 12%–15% of BV’s dry weight (Wehbe et al., 2019; Carpena et al., 2020). It hydrolyzes the sn-2 fatty acyl ester bond of membrane glycerol-3-phospholipids to liberate fatty acids and lysophospholipids, and this catalytic activity disrupts cell membranes, contributing to its anti-tumor, anti-infectivity, and other effects (Putz et al., 2007; Carpena et al., 2020). Besides, the abundant amino acids in bvPLA_2_, leucine and lysine, promotes the phenomenon of neurotoxicity (Pattabhiramaiah et al., 2020).

### Apamin

Apamin, an 18 amino acid peptide, makes up 2%–3% of its total dry weight (Gu et al., 2020). It is formed by a disulfide bond between two cysteines, which shapes its highly stable and compact chemical structure (Nguyen et al., 2015). Apamin has demonstrated the potential benefits in anti-atherosclerosis, anti-heart failure, and improvement of neurological disorders (Gu et al., 2020).

## Anticancer effects

**FIGURE 2:**
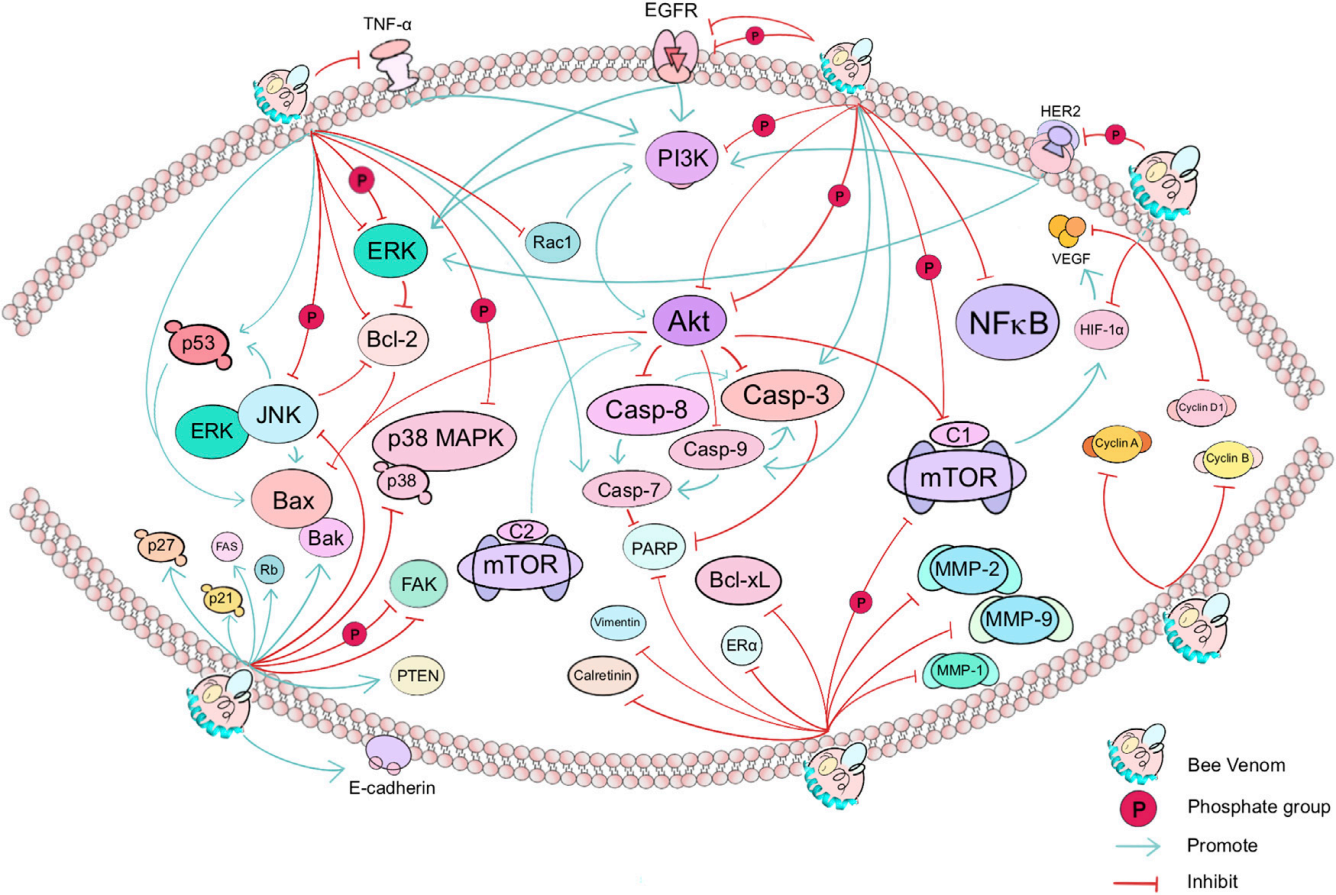
The main affected targets and pathways in anticancer effects of BV. In cancer, BV mainly affects the PI3K/Akt/mTOR pathway (e.g., PI3K, Akt, and mTOR), apoptosis signaling pathway (e.g., EGFR and TNF-α, including downstream effectors such as Casp-3, Casp-7, Casp-8, Casp-9, Bcl-2, Bax and Bcl-xL), p38 MAPK pathway, and thus affect the growth, differentiation, invasion, autophagy or migration of cancer cells in lung, breast, cervical and other cancers. Green arrows or red cut-off lines represent the “promote” or “inhibit” effect of the target (gene or protein) by the upstream target factor, respectively. Bee Venom, known as BV, is dispersed on the surface of a phospholipid bilayer. The text shows the direct or indirect targets of BV.

The incidence rate of cancer, the most serious cause of death, is constantly testing the global medical system’s coping and resolving ability (Sung et al., 2021). The morbidity of many cancers, e.g., lung cancer, breast cancer, and colorectal cancer is still high, and the exploration of various forms, approaches and strategies of cancer treatment is still serious (Siegel et al., 2021). Animal-derived venoms are rich in a large number of active proteins and enzymes and have potential anticancer activities (Ejaz et al., 2018). As a promising natural product, BV and its main component MEL can regulate the cell cycle, change the permeability of cell membrane, inhibit the proliferation and migration, and promote endogenous/exogenous apoptosis and autophagy and other regulatory cell death modes to promote cell death (Mirzaei et al., 2021). Thus, it shows potential in strategies for inhibiting the occurrence and development of cancer and tumor (Wehbe et al., 2019; Carpena et al., 2020), as shown in Table 1, and the main affected targets and pathways in anticancer effects of BV is shown in Figure 2.

**TABLE 1:** Summary of the anti-cancer effects and mechanisms of BV and its main components.

BV and/or its components	Model	Cell/Animal (BV etc. administration)	Effects	Mechanisms	Reference
Lung cancer					
BV	Lung cancer	A549 (EGF induce; 0.1, 0.5, 1 and 2 μg/ml, for 1, 2, 12 and 24 h)	Inhibit cell migration and invasion; inhibit EMT	↓F-actin recombination; ↓EMT: ↑E-cadherin, ↓vimentin, ↓ZEB2, ↓Slug; ↓MARK pathway: ↓p-ERK, ↓p-JNK; ↓mTOR pathway: ↓p-FAK, ↓p-mTOR, ↓p-p70S6K, ↓p-4E-BP1	Jeong et al. (2019)
		H1739, H23 (EGF induce; 0.1, 0.5, 1 and 2 μg/ml, for 12 h)	Inhibit EMT	↓F-actin recombination; ↓EMT: ↑E-cadherin, ↓vimentin	
MEL	Lung cancer	A549, H358 (2 μg/ml, for 24, 48 and 72 h)	Inhibit cell growth, migration and invasion; induce apoptosis	↑Caspase-3, ↑Apaf-1; ↓ERK signaling pathway: ↓TGF-β, ↓ERK, ↓p-ERK	Yu et al. (2020)
		Male nude mice (5 mg/kg, local injection, once every 3 days for 21 days)	Inhibit tumor growth, prolong the survival time of mice	↓TGF-β, ↓ERK	
MEL	Lung cancer	ChaGo-K1 (0.7 and 2.5 μM, for 24 h)	Cell shrinkage and floating; inhibit cell proliferation and migration; promote apoptosis/necrosis; G0/G1 phase arrest	↑Bcl-2, ↓MADD	Tipgomut et al. (2018)
MEL	Lung cancer	A549/DDP (2, 4 and 8 μg/ml, for 48 h)	Inhibit the Warburg effect; inhibit cell growth and induce apoptosis	↓Tripartite motif-containing 8 (TRIM8); Akt pathway: ↓p-Akt	Zhang et al. (2021)
		Balb/c athymic nude mice (2 mg/kg, i.p., every 7 days for 3 weeks)	Inhibit tumor growth and reduce tumor size and mass; enhance the sensitivity of tumor and cells to cisplatin	—	
MEL	Lung cancer	NCI-H441 (0.5, 1, 2 and 4 μg/ml, for 24, 48 and 72 h)	Promote cell apoptosis, inhibit migration and invasion; inhibit cell growth	↓miR-183, ↓Bcl-2, ↑Caspase-2, ↑Bax	Gao et al. (2018)
		A549 (2 μg/ml, for 72 h)	Inhibit cell growth	—	
		Balb/c nu/nu mice (5 mg/kg, s.c., daily treated for 4 weeks)	Inhibit tumor growth	↑Caspase-2	
MLT@ZIF-8 NPs	Lung cancer, cervical carcinoma	A549 (2, 4 and 8 μg/ml, for 24 h)	Cells become smaller and round, chromatin condenses and nucleus shrinks; inhibit cell activity; promote apoptosis and reduce hemolysis	↑p53, ↑Bax, ↑Cyt C, ↑Caspase-3, ↑Caspase-9, ↓p-Akt, ↓PI3K, ↓Bcl-2	Li et al. (2018)
		HeLa (1, 2, 4, 6 and 8 μg/ml, for 24 h)	Inhibit cell activity	—	
		U14 tumor-bearing Kunming mice (MEL containing 1 mg/kg, i.v., daily treated for 3 days)	Inhibit tumor growth	—	
MpG@LPN	Lung cancer	A549 (5, 10, 15, 20 and 25 μg/ml, for 3, 24 and 48 h)	Induce apoptosis; reduce hemolysis and nonspecific cytotoxicity	↑Caspase-3	Ye et al. (2021)
		Female athymic nude mice (5 mg/kg, i.v., every other day for 5 times)	Inhibit tumor growth and reduce liver injury; improve tumor targeting ability	—	
Breast cancer					
BV, MEL, MEL and docetaxel	Breast cancer	SUM159 (2.5, 5, 5.58, 10, 15 and 20 ng/μL (BV), 2.5, 4.24, 5, 10, 15 and 20 ng/μL (MEL), for 1, 18 and 24 h)	The plasma membrane shrinks and is destroyed; reduce cell viability, induced cell death/apoptosis	PI3K/Akt and MAPK signaling pathway: ↑cleaved Capsase-3, ↑p-p44/42 MAPK, ↑p-Akt, ↓p-EGFR (BV).	Duffy et al. (2020)
				PI3K/Akt and MAPK signaling pathway: ↑cleaved Capsase-3, ↑p-p44/42 MAPK, ↑p-stress-activated protein kinase (SAPK)/JNK, ↑p-p38 MAPK, ↓p-EGFR, ↓p-Akt (MEL)	
		SKBR3 (2.5, 5, 5.77, 10, 15 and 20 ng/μL (BV), 2.5, 5, 10, 15 and 20 ng/μL (MEL), for 1 and 24 h)	Reduce cell viability	PI3K/Akt and MAPK signaling pathway: ↓p-HER2, ↓p-EGFR, ↓p-p44/42 MAPK, ↓p-Akt, ↓p-SAPK/JNK, ↓p-p38 MAPK	
		MDA-MB-231 (2.5, 5, 10, 15 and 20 ng/μL (MEL), for 1 h)	—	↓p-EGFR, ↓p-MARK (MEL)	
		BALB/cJ female mice (5 mg/kg (MEL) and/or 7 mg/kg (docetaxel), intratumoral injection, on days 3, 5, 7, 9, 11, 13 and 15 post inoculation of T11 cells)	Reduce tumor cell proliferation; enhance cell sensitivity to docetaxel (MEL)	↓PD-L1, ↓p-EGFR, ↓p-HER2 (MEL)	
BV	Breast cancer, hepatocellular carcinoma	MDA-MB-231 (8, 12, 25, 50 and 100 μg/ml, for 45 min and 24 h)	Induce nuclear reduction; reduce cell viability; reduce mitochondrial membrane permeability; reduce 5meC and 5hmC; make 5 fC and 5caC increase first and then decrease	↓DNA methylation	Uzuner et al. (2021)
		HepG2 (8, 12, 25, 50 and 100 μg/ml, for 45 min and 24 h)	Reduce cell viability; reduce mitochondrial membrane permeability; increase mtDNA CNV; make 5meC, 5hmC, 5 fC and 5caC increase first and then decrease	↑DNA methylation	
BV	Breast cancer, lung cancer	MCF-7, A549 (for 24 h)	Inhibit cell proliferation; G1 phase arrest	↑H_2_O_2_; ↓Cyclin D1, ↓The nuclear translocation of NF-κB	Yoon et al. (2018)
MEL	Breast cancer	4T1, MCF-7	Inhibit cell viability	↑Bax/Bcl-2	Chang et al. (2020)
		Mice (i.p.)	Improve tumor radiosensitivity; inhibit tumor growth	—	
MEL	Breast cancer	4T1 (0.125, 0.25, 0.5, 1, 2, 4, 8, 16 and 32 μg/ml, for 72 h)	Inhibit cell proliferation promote cell hemolysis and apoptosis	↑Mfn1, ↑Drp1	Moghaddam et al. (2020)
MEL	Breast cancer	MDA-MB-231 (5% O_2_ induce; 1, 2 and 4 μg/ml, for 6 and 24 h)	Inhibit cell proliferation and induce apoptosis; adjust TME	↑Bax, ↑TNF-α ↓HIF-1α signaling pathway: ↓NF-κB, ↓HIF-1α, ↓LDHA, ↓VEGFA	Mir Hassani et al. (2021)
BV, MEL	Breast cancer	MCF-7 (0.1, 1, 10, 50 and 100 μg/ml (BV), 0.1, 1, 5, 10, 25 μg/ml (MEL), for 24 h)	Inhibit cell growth	—	Lischer et al. (2021)
BV (combined with hesperidin, piperine, tamoxifen)	Breast cancer	MCF-7, T47D (at a non-constant combination concentration, for 24 h)	Reduce cell viability and induce apoptosis; G2/M phase arrest	↑Bax, ↓Bcl-2, ↓ERα, ↓EGFR	Khamis et al. (2018)
BV, BV and *Annona muricata* fruit	Breast cancer	Wistar albino rats (N-methylnitrosourea induce; 75 μg/kg (BV), s.c., 2 dose of BV on the 4th and 16th day of gestation, 200 mg/kg (*A. muricata* fruit), from day 4 of pregnancy till weaning, 75 μg/kg (BV) and 200 mg/kg (*A. muricata* fruit))	Restore ovarian tissue structure and damage	↓MDA, ↑CAT, ↑SOD; ↓MMP-1, ↓NF-κB, ↓TNF-α, ↓p53, ↓Calretinin, ↑Caspase-3	El-Beltagy et al. (2021)
BV, BV and cisplatin	Breast cancer	4T1 (2, 4, 6, 8 and 10 μg/ml (BV), 5, 10, 15, 20, 25 and 30 μg/ml (cisplatin), 2, 4, 6, 8 and 10 μg/ml (BV) and 10 μg/ml (cisplatin), for 24 h)	Reduce cell viability and induce death; promote the cytotoxicity of cisplatin to cells	—	Arani et al. (2019)
MEL, MEL and plasma-treated phosphate buffered saline solution	Breast cancer, skin cancer (melanoma)	MCF-7, A375 (0.5, 1, 1.5 and 2 μg/ml (MEL), 0.5, 1, 1.5 and 2 μg/ml (MEL) and 10% (plasma-treated phosphate buffered saline solution), for 24 h)	Reduce cell viability; induce late apoptosis/necrosis and lipid peroxidation; inhibit tumor growth, reduce tumor size and mass	—	Shaw et al. (2019)
MEL loaded on FA-PENs	Breast cancer	MDA-MB-231 (15 μg/ml, for 24 and 48 h)	Lead to apoptosis and cell cycle arrest	↓Cyclin D1, ↓Bcl-2, ↓Caspase-8	Motiei et al. (2021)
MEL loaded on nGO, GN and ND	Breast cancer	MCF-7, MDA-MB-231 (10 mg/L (MEL), 20 mg/L (MEL) and 10 mg/L (GN), 20 mg/L (MEL) and 10 mg/L (nGO), 20 mg/L (MEL) and 10 mg/L (ND), for 24 h)	The cell bodies shriveled and cell protuberances were shortened; reduce cell metabolic activity; induce cell necrosis and apoptosis	↑ROS; ↑Bax, ↑high temperature requirement protease A (HTRA), ↑Caspase-3, ↑Caspase-8, ↓p21, ↓XIAP	Daniluk et al. (2020)
MEL loaded on niosome	Breast cancer	4T1 (72.42 μM (MEL), 97.41 μM (melittin-loaded niosome), for 72 h); SKBR3 (65.13 μM (MEL), 85.76 μM (melittin-loaded niosome), for 72 h)	Reduce cell viability; promote cell apoptosis, inhibit migration and wound healing	↑Caspase-3, ↑Caspase-9, ↑Bax, ↓MMP-2, ↓MMP-9, ↓Bcl-2	Moghaddam et al. (2021)
		Female BALB/c inbred mice (3 and 6 mg/kg (MEL), 1.5 and 3 mg/kg (melittin-loaded niosome), i.p., daily injection for 20 days)	Inhibit tumor growth, reduce tumor volume and the number of inflammatory cells in tumor; inhibit weight loss in mice	—	
MEL loaded on CA-MNPs	Breast cancer	MCF-7 (0.097, 0.195, 0.39, 0.781, 1.56, 3.125, 6.25 and 12.5 μg/ml, for 48 h)	Inhibit cell growth; improve the ability of magnetic targeting tumor	—	Hematyar et al. (2018)
MEL loaded on APNPs	Breast cancer	MDA-MB-231 (0.25, 0.5 and 1 μM, for 3 days)	Inhibit cell growth	—	Yu et al. (2018)
		Tumor-bearing mice (24 μg per mouse, i.v., 3 times a week for 2 weeks)	Promote tumor cell apoptosis; inhibit the tumor growth; improve tumor targeting ability	—	
MEL loaded on PIC	Breast cancer	MCF-7 (15 and 30 μg/ml, for 3, 7 days)	Enhance the uptake and the cytotoxicity of MEL; inhibit cell growth; reduce hemolysis and prevent MEL degradation; improve tumor targeting ability	—	Raveendran et al. (2020)
Cervical cancer					
BV	Cervical cancer	HeLa, Caski (1, 5 and 10 μg/ml, for 12, 24 and 48 h)	Reduce cell viability; inhibit cell proliferation, migration and wound healing; promote cell death and apoptosis	↑p21, ↑p27, ↑p53, ↑Rb, ↑Bax, ↑cleaved PARP, ↓Cyclin A, ↓Cyclin B, ↓Bcl-2, ↓Bcl-xL, ↓HPV E6, ↓HPV E7, ↓pro-Caspase-3, ↓pro-Caspase-9; ↓Mitotic signaling pathway: ↓Akt, ↓p-Akt, ↓JNK, ↓p-JNK, ↓p38, ↓p-p38, ↓p44/42, ↓p-p44/42	Da-Hyun Kim et al. (2020)
BV	Cervical cancer, lung cancer, breast cancer	HeLa (12.5 μg/ml, for 12 h); A549 (3.125 and 12.5 μg/ml, for 12 h); MDA-MB-231 (6.24 and 12.5 μg/ml, for 12 h)	Cell contraction, irregular character and cell membrane damage; reduce cell viability and induce apoptosis	—	Borojeni et al. (2020)
MEL	Cervical cancer	HeLa (1, 1.8 and 4 μg/ml, for 12 h)	Cell shrinkage, cytoplasm condensation, cell structure became disorder and lost shape; inhibit cell proliferation and induce apoptosis	—	Zarrinnahad et al. (2018)
MEL loaded on PEG-GO-Fe_3_O_4_	Cervical carcinoma	HeLa (13 μg/ml (PEG-GO-Fe_3_O_4_/MEL), containing 5 μg/ml (MEL), for 24, 48 and 72 h)	Prevent MEL from denaturation or degradation; induce cell contraction, deformation and membrane rupture; inhibit cell growth and promote apoptosis	—	Qi et al. (2020)
BV loaded on NFC	Cervical carcinoma	HeLa (500 μg/ml, for 24 and 48 h)	Cell contraction and cell membrane blistering; promote apoptosis	—	Alalawy et al. (2020)
Pancreatic cancer, gastric cancer, and colorectal cancer					
MEL	PDAC	PDAC cells (SW 1990, etc. 3 μg/ml, for 48 h)	Inhibit cell growth, migration, wound healing and EMT	↑NONHSAT105177; ↓EMT pathway: ↓Snail, ↓Slug, ↓vimentin, ↑E-cadherin	Wang et al. (2018)
MEL	Gastric cancer	AGS (0.05, 0.1 and 0.15 μM, for 24 h)	Reduce cell viability; inhibit cell migration, invasion and EMT; inhibit cell adhesion and colony formation	↓MMP-2, ↓MMP-9, ↓MMP-13; PI3K/Akt signaling pathway: ↓p-Akt, ↓PI3K; ↓Wnt/β-catenin signaling pathway: ↓Wnt-5α, ↓β-catenin, ↓vimentin, ↓N-cadherin, ↑E-cadherin; BMP/Smad signaling pathway: ↓Smad 1/5/8, ↓BMP, ↑glycogen synthase kinase 3α/β (GSK3 α/β)	Huang et al. (2021)
BV, MEL	Colorectal cancer	HCT-116, SW-480 (1, 5 μg/ml, for 24 h)	Reduce cell viability; induce early and late apoptosis; affect the biotransformation of cancer cell	↑Mitochondrial apoptosis pathway: ↑Fas, ↑Caspase-9; ↓CYP1A1, ↓GSTP1, ↓Bcl-2, ↓Bax (except HCT-116 with BV), ↑MRP-2 (HCT-116 with MEL), ↓MRP-2 (SW-480 with MEL)	Nikodijevic et al. (2021)
MEL	Colorectal cancer, gastric cancer	COLO205, HCT-15, AGS (5, 10 and 20 μg/ml, for 30 s, 1, 5, 10, 15 min and 4 h)	Cytoplasmic contraction and cell membrane damage; inhibit cell growth and induce cell death	—	Soliman et al. (2019)
BV, MEL, bvPLA_2_	Colorectal cancer	HCT116 (14.05 μg/ml (MEL), 10 and 50 μg/ml (bvPLA_2_), for 24 h)	Reduce cell viability and inhibit proliferation (BV, MEL); MEL and bvPLA_2_ synergistically inhibited cell proliferation	—	Yaacoub et al. (2021)
BV	Colorectal cancer	HT-29 (7.5, 15 and 30 μg/ml, for 24 and 48 h)	Inhibit cell proliferation and promote apoptosis	↑15-lipoxygenase-1	Zare et al. (2019)
MEL loaded on oligopeptide alginate NPs	Human clonal colon adenocarcinoma	Caco-2 (2.5 and 5 μM, for 48 h)	Induce cell death	—	Wattanakul et al. (2019)
Hepatocellular carcinoma					
MEL	Hepatocellular carcinoma	HepG2 (3, 5 and 10 μg/ml, for 4, 8, 12, 24, 48 and 72 h)	Inhibit cell growth, induce apoptosis and autophagy	↓Bcl-2, ↓p62, ↑Beclin 1, ↑LC3; ↑Mitochondrial apoptotic pathway (When autophagy is inhibited): ↑Cyt C, ↑Caspase-3, ↑Caspase-9	Lv et al. (2019)
MEL	Hepatocellular carcinoma	SMMC-7721 (CoCl_2_ induce; 2 and 4 μg/ml, for 24 h)	Inhibit the formation of EMT and VM; reduce cell viability; inhibit cell migration and invasion	↓N-cadherin, ↓vimentin, ↑ E-cadherin, ↓VEGF, ↓MMP-2, ↓MMP-9; ↓Akt pathway: ↓HIF-1α, ↓p-Akt	Chen et al. (2019)
		Huh7, HepG2 (2 and 4 μg/ml, for 24 h)	Inhibit the formation of VM; reduce cell viability; inhibit cell migration and invasion	↓VEGF, ↓MMP-2, ↓MMP-9, ↓HIF-1α	
		BALB/c nude male mice (50 and 100 μg/kg, i.v., daily injection for 11 days)	Inhibit tumor growth and reduce tumor volume	↓HIF-1α	
BV, MEL, BV and sorafenib; MEL and sorafenib	Hepatocellular carcinoma	HepG2 (at a non-constant combination concentration, for 24 h)	Reduce cell viability and inhibit proliferation; G2/M phase arrest	↑MDA; ↑p53, ↑Bax, ↑Caspase-3, ↑Caspase-7, ↑PTEN, ↓Bcl-2, ↓Cyclin D1, ↓HIF-1α, ↓VEGF, ↓Rac1, ↓MMP-9, ↓NF-κB	Mansour et al. (2021)
Bladder cancer					
MEL	Bladder cancer	T24, 5637 (4 μg/ml, for 48 h)	Inhibit cell proliferation and migration	↓PI3K/Akt pathway: ↓LPAR1, ↓COL5A1, ↓COL6A2; ↓TNF pathway: ↓CXCL1, ↓CXCL2, ↓CXCL3	Jin et al. (2018)
MEL	Bladder cancer	UM-UC-3, 5637 (2, 4 and 6 μg/ml, for 24 h)	Inhibit cell proliferation, migration and invasion	↓MAPK pathway: ↓ERK5, ↓MEK5, ↓ERK1/2, ↓p-ERK1/2, ↓JNK, ↓p-JNK, ↓p-p38, ↓NRAS, ↓PAK1, ↓PAK2, ↓EGFR; ↓V-ATP: ↑ATP6V1F, ↓ATP6V0B, ↓ATP6V1C1, ↓ATP6V1E2, ↓ATP6V0C, ↓ATP6V0A2	Yao et al. (2020)
Skin cancer					
BV, MEL, MEL and temozolomide	Melanoma	A375SM (1, 2.5 and 5 μg/ml (BV, MEL), 50 μM (temozolomide), 1, 2.5 μg/ml (MEL) and 50 μM (temozolomide), for 24 and 72 h)	Inhibit cell growth, migration, invasion and promote apoptosis	↑cleaved Caspase-3, ↑cleaved Caspase-9, ↓MITF; ↓PI3K/Akt/mTOR pathway: ↓p-PI3K, ↓p-Akt, ↓p-mTOR; ↓MAPK signaling pathway: ↓ERK,↓p-ERK, ↓p38, ↓MMP-2	Lim et al. (2019)
		B16F10 (0.5, 1, 2.5 and 5 μg/ml (BV, MEL), for 24, 72 h); SK-MEL-28 (1, 2.5 and 5 μg/ml (BV, MEL), 50 μM (temozolomide), 1, 2.5 μg/ml (MEL) and 50 μM (temozolomide), for 24 and 72 h)	Inhibit cell growth, migration, invasion and promote apoptosis; inhibit melanin production	—	
MEL-AF	Melanoma	A375 (1, 2 and 3 μg/ml, for 2 and 24 h)	Inhibit cell proliferation, migration and invasion, and induce apoptosis	↑F-actin, ↓EGFR (MEL-AF); ↑Mitochondrial pathway: ↑Cyt C, ↑Caspase-3, ↑Caspase-9	Sangboonruang et al. (2020)
MEL, MEL and 5-fluorouracil	Skin squamous cell carcinomas	A431 (0.52 μM (MEL), 0.25 μM (5-fluorouracil), 0.52 μM (MEL) and 0.25 μM (5-fluorouracil), for 72 h)	Cell abscission and contraction, DNA fragmentation; inhibit cell proliferation and induce apoptosis/necrosis; S and G2/M phase arrest; enhance the sensitivity of cells to 5-fluorouracil	—	Ombredane et al. (2021)
BV, BV and cisplatin	HNSCC	UMSCC12, UMSCC29, UMSCC38, UMSCC47 (at a non-constant combination concentration, for 24 h)	Reduce cell viability and inhibit growth; G2/M phase arrest	↑Bax, ↓Bcl-2, ↓EGFR	Grawish et al. (2020)
Leukemia					
MEL	Leukemia	CCRF-CEM, K-562 (1, 10 and 100 μM, for 24 and 48 h)	Reduce cell viability and induce apoptosis	↑Caspase-3/7 pathway: ↑hydrolytic activity of Caspase-3/7	Ceremuga et al. (2020)
MEL	Leukemia	Jurkat (10^−5^ M, for 0.5 h)	Inhibit cell survival; increase permeability through the plasma membrane	—	Gasanoff et al. (2021)
Melectin	Leukemia	K562 (10, 20, 30 and 40 μM, for 0.5 and 4 h); K562/ADM, HL-60, Jurkat (10, 20, 30 and 40 μM, for 4 h)	Destroy cell membrane; inhibit cell proliferation	↑LDH	Liang et al. (2021)
Other tumors					
MEL	Hodgkin lymphoma	KM-H2 (0.5, 1 and 1.5 μM, for 24 and 72 h)	Inhibit cell survival;	—	Kreinest et al. (2021)
		L-428 (0.5, 1 and 1.5 μM, for 24 and 72 h)	Inhibit cell survival; enhance the sensitivity of cells to cisplatin		
MEL	Human osteosarcoma	143B (1, 2 and 4 μg/ml, for 24 and 48 h)	Inhibit cell invasion; inhibit cell and tumor migration	↓Wnt/β-catenin signal pathway: ↓MMP-2, ↓MMP-9, ↓Cyclin D, ↓LRP5, ↓β-catenin, ↓C-myc, ↓survivin, ↓VEGF	Zhu et al. (2021)
		Female BALB/c^nu/nu^ nude mice (160, 320 and 640 μg/kg, intratumoral injection, each course lasted for 5 days and lasted for 4 courses, with an interval of 1 day between the 2 courses)	Reduce tumor size and mass; reduce the number of pulmonary metastatic nodules		
BV, MEL	Glioblastoma multiforme	Hs683, T98G, U373 (2.5, 5 and 10 μg/ml, for 1, 4, 8 and 72 h)	Reduce cell viability; promote late apoptosis and necrosis	↑Bak, ↑Bax, ↓Caspase-3; Long non-coding RNAs: ↑RP11-838N2.4, ↑XIST	Lebel et al. (2021)
BV, MEL; BV and γ-radiation; MEL and γ-radiation	Ehrlich ascites carcinoma	EAC cell (30, 60, 120, 240, 480 and 960 μg/ml, for 24 h)	Inhibit cell viability	—	El Bakary et al. (2020)
		Female albino tumor-bearing mice (0.56 mg/kg (BV), 500 μg/kg (MEL), i.p., daily for 21 days, 0.5 Gy (γ-radiation) each time for 30 min)	Reduce tumor volume; destroy tumor tissue	↑CAT; ↓TNF-α, ↓VEGF-A, ↓MMP-2, ↓MMP-9, ↑Caspase-3	
MEL loaded on ^9^G-A7R-Disk	Glioma	U87 (1, 2 and 4 μM, for 72 h)	Inhibit cell growth	—	Wang et al. (2019)
		Male BALB/c nude mice (total does of 18 mg/kg, i.v., at the 6th, 8th, 10th, 12th and 14th day)	Promote tumor cell apoptosis and inhibit angiogenesis; cause tissue damage		

Abbreviationsare as shown in the literature. (↓), down-regulation or inhibition; (↑), up-regulation or activation.

### Anti-lung cancer research

In 2020, Lung cancer became the second most common malignancy worldwide with an incidence rate of 11.4% (Sung et al., 2021), mainly non-small cell lung cancer, occupying 80% of all new lung cancer cases (Sugarbaker and Dasilva, 2011). BV could inhibit epithelial-mesenchymal transition (EMT), increase the expression of vimentin, down-regulate the E-cadherin expression, and inhibit the recombination of F-actin related to the tumor metastasis in lung cancer A549, H1739 and H23 cells induced by epidermal growth factor (EGF). In A549 cells, BV decreased the phosphorylation of extracellular-regulated kinase (ERK), c-Jun N-terminal kinase (JNK), focal adhesion kinase (FAK) and mammalian target of rapamycin (mTOR), and eventually provoked the decrease of the transcription factors zinc finger E-box-binding homeobox 2 (ZEB2) and Slug in the EMT, suggesting its potential function in anti-cancer cell metastasis (Jeong et al., 2019). At the same time, as the main component of BV, MEL had a blockade effect on transforming growth factor-β (TGF-β), ERK and phosphorylated ERK in the ERK signaling pathway, resulting in the synthesis of Caspase-3 and Apaf-1 proteins that promoted apoptosis in A549 cells, and the cell growth, migration, invasion and other activities were blocked (Yu et al., 2020). Similarly, MEL also increased the apoptotic ratio in ChaGo-K1 of lung cancer, and the expression of mitogen activating protein-kinase activating death domain (MADD) decreased, which further brought about cell cycle arrest in G0/G1 phase (Tipgomut et al., 2018).

After cisplatin-resistant lung cancer cells A549/DDP were cultured *in vitro* and treated with MEL, the Warburg effect as well as phosphorylated protein kinase B (Akt) were suppressed; after vaccinating A549/DDP into Balb/c athymic nude mice and treating them via intraperitoneal (i.p.) injection of MEL, their tumor and cell sensitivity to cisplatin was enhanced and tumor size and mass were controlled (Zhang et al., 2021). In addition, the miR-183 played a role as a tumor marker of lung cancer, and was inhibited by MEL in NCI-H441 cells. Its inhibition further increased the expression of Caspase-2 and Bcl-2-associated X protein (Bax), and reduced the Bcl-2 expression. Not only that, after subcutaneous (s.c.) injection of MEL in Balb/c nu/nu mice, Caspase-2 was elevated and tumor growth was restricted similarly (Gao et al., 2018).

Besides, MEL-carried nanoparticles (NPs) systems have considerably enhanced the security of MEL *in vivo* and its efficacy against tumors, offering the possibility of tumor elimination (Zhou et al., 2021). Such drug nano-delivery platforms have been observed in lung cancer research. MEL-carried zeolitic imidazolate framework-8 (MLT@ZIF-8) NPs (Li et al., 2018) and lipid-coated polymer NP (MpG@LPN) (Ye et al., 2021) increased apoptosis in A549 cells and inhibited tumor growth. In the meanwhile, the cellular hemolysis caused by piggybacking on these two nanomaterials was reduced to a certain extent compared with MEL alone.

### Anti-breast cancer research

Breast cancer, as the most commonplace tumor in female population, has a very strong time variant tumor metastasis and spatial heterogeneity associated with genotype and phenotypic differences, leading to a continuous change in the evaluation and treatment process of breast cancer (Fumagalli and Barberis, 2021).

BV and MEL induced strongly selective cell death in triple-negative breast carcinoma and human epidermal growth factor receptor 2 (HER2) enriched breast cancer with little effect in routine cells, through interfering with growth factor-dependent receptor tyrosine kinase interactions critical for receptor phosphorylation and activation of phosphoinositide 3-kinase (PI3K)/Akt and mitogen-activated protein kinase (MAPK) signaling. Besides, in an allograft model, the effect of docetaxel in suppressing breast tumor growth was potentiated by the administration of MEL, and the programmed death ligand-1 (PD-L1) protein expression, phosphorylated HER2 and epidermal growth factor receptor (p-EGFR), were significantly reduced (Duffy et al., 2020). Researchers also investigated the effect of BV on epigenetic changes in cancer cells: after confirming that BV decreased the viability and mitochondrial membrane permeability of MDA-MB-231 cells, they further inspected epigenetic and mitochondrial DNA (mtDNA) Copy Number Variation (CNV). The experimental results revealed that BV generated morphological changes in the nucleus of MDA-MB-231 cells, and the exploration of cytosine modification in cancer cells showed that 5′-methylcytosine (5meC), 5′-hydroxymethylcytosine (5hmC) cells decreased rapidly after treatment with BV in MDA-MB-231 cells. And 5′-formlylcytosine (5 fC) and 5′-carboxycytosine (5caC) exhibited a similar increasing and then decreasing process (Uzuner et al., 2021).

BV reduced the expression of nuclear translocation of nuclear factor-κB (NF-κB) and Cyclin D1, enhanced H_2_O_2_ production, blocked G1 cycle and inhibited breast cancer proliferation in MCF-7 cells (Yoon et al., 2018). MEL reduced the viability of 4T1 and MCF-7 cell lines, and the addition of irradiation resulted in a significant increase of Bax/Bcl-2 value. Besides, MEL enhanced tumor radiosensitivity and inhibited the tumor growth in 4T1 tumor-bearing mice (Chang et al., 2020). MEL also showed the potential to promote mitofusin-1 (Mfn1) and dynamin-related protein 1 (Drp1) expression and apoptosis in 4T1 cells (Moghaddam et al., 2020). After 5% O_2_ induction in MDA-MB-231 cells, the hypoxia-inducible factor-1α (HIF-1α) signaling pathway was inhibited by MEL and the expression of NF-κB, HIF-1α, vascular endothelial growth factor A (VEGFA) and lactate dehydrogenase A (LDHA) decreased, while the expression of Bax and tumor necrosis factor-α (TNF-α) was reversed, ultimately disrupting the tumor microenvironment (TME) of cancer cells and activating the phenomenon of apoptosis (Mir Hassani et al., 2021).

BV alone and in combination with other drugs or solutions had an anti-breast cancer impact (Khamis et al., 2018; Arani et al., 2019; El-Beltagy et al., 2021). *In vitro*, BV inhibited the growth of MCF7 and T47D cells, while blocking the cell cycle in the G2/M phase. BV alone or in combination with tamoxifen, hesperidin, and piperine resulted in reduced expression of Bcl-2, EGFR, and estrogen receptors α (ERα) receptors, and elevated expression of Bax, which eventually led to apoptosis. The anticancer and anti-drug resistance effects of tamoxifen were enhanced by the synergistic effect of BV (Khamis et al., 2018). *In vivo*, N-methylnitrosourea was able to induce breast cancer and ovarian complications in Wistar albino rats. After the combination of BV and *Annona muricata* fruit, serum levels of matrix metalloproteinase-1 (MMP-1), NF-κB, TNF-α, malondialdehyde (MDA), elevated Caspase-3, superoxide dismutase (SOD), catalase (CAT), and ovarian histopathological changes due to mammary carcinoma were improved in mothers and offspring rats. Additionally, calreticulin and p53 protein response in the ovarian stroma switched from positive to negative (El-Beltagy et al., 2021). In addition, the non-specific cytotoxicity of MEL in the clinical setting cannot be conveniently ignored. However, plasma-treated phosphate buffered saline solution can cause the death of MCF-7 and A375 melanoma cells on the one hand and circumvent the non-specific cytotoxicity of MEL to a certain extent on the other, revealing the value of the combination therapy (Shaw et al., 2019).

The application of nanomaterials in combination with MEL has also yielded beneficial results in the treatment of breast carcinoma. The disruption of cell membrane by MEL was unaffected by loading in the NPs after carrying the MEL, and its effect on causing necrosis or apoptosis of the tumor or cancer cells remained undisturbed, such as folic acid (FA)-polyelectrolyte nanocarriers (PENs) (Motiei et al., 2021), nanographene oxide (nGO) and graphene (GN) (Daniluk et al., 2020), the niosome (Moghaddam et al., 2021), citrate functionalized Fe_3_O_4_ magnetic NPs (CA-MNPs) (Hematyar et al., 2018), and activatable protein NPs (APNPs) (Yu et al., 2018). Besides, after carrying MEL in poly-ion complex (PIC) added with estrone, it prevented the degradation of MEL in cells and increased the uptake of MEL and cytotoxicity (Raveendran et al., 2020).

### Anti-cervical cancer research

Cervical cancer is one of the most common cancers in the global female population (Volkova et al., 2021). BV demonstrated the ability to inhibit the growth and migration of HPV-positive cervical cancer Caski and HeLa cells, and cell cycle protein Cyclin A and Cyclin B, Akt, JNK and p38/44/42 and their phosphorylated proteins associated with mitogenic signaling pathways were inhibited, and pro-Caspase-3, pro-Caspase-9, cleaved polyadenosine-diphosphate-ribose polymerase (PARP), Bcl-2 and Bcl-xL expression was reduced. On the contrary, proteins such as p53, p21, and retinoblastoma (Rb) were upregulated in expression with the utility of BV, and the number of dead and apoptotic cells was significantly promoted (Kim D. H. et al., 2020). It is worth noting that the main mode of death of HeLa cells after BV treatment is apoptosis, which causes severe cell membrane damage and cell shrinkage (Borojeni et al., 2020), while MEL can show the effect of inhibiting HeLa cell proliferation and inducing apoptosis such as cell shrinkage and structural disorganization (Zarrinnahad et al., 2018).

Graphene oxide magnetic nanocomposites (PEG-GO-Fe_3_O_4_)/MEL complexes caused time-dependent toxic effects on HeLa cells with deformation lysis, membrane breakage and other abnormal cellular states. The experimental results demonstrated that this material achieved the long-lasting release and effect enhancement of MEL, while preventing the degradation or denaturation of MEL, ensuring the anti-cervical cancer effect of MEL (Qi et al., 2020). BV loaded on nano-fungal chitosan (NFC) also showed effective anticancer activity in promoting apoptosis in HeLa cells (Alalawy et al., 2020).

### Anti-pancreatic cancer, gastric cancer, and colorectal cancer research

BV and MEL have also been used to treat cancers of the digestive system, such as pancreatic cancer, gastric cancer, and colorectal cancer.

In pancreatic ductal adenocarcinoma (PDAC), overexpression of NONHSAT105177 in long non-coding RNAs is associated with activities such as cell proliferation and migration (Wang et al., 2018). This RNA is able to increase its expression under the regulation of MEL, further promoting its inhibitory effect on PDAC, which is related to EMT pathway-related proteins, such as causing the repressive expression of Snail, Slug and vimentin and the up-regulated expression of E-cadherin.

MEL exhibited inhibitory effects on human gastric cancer AGS cell viability, adhesion, colony-forming ability, EMT, and a limiting effect on MMP-2, MMP-9 and MMP-13 proteins related to cell migration and invasion ability. In addition, MEL tended to act more in a variety of signaling pathways, containing bone morphogenetic protein (BMP)/Smad, Wnt/β-catenin and PI3K/Akt pathways (Huang et al., 2021).

When BV and MEL were applied to HCT-116 and SW-480 of colorectal cancer cells, respectively, the mitochondrial apoptotic pathway was activated, cancer cell viability was reduced, chromatin was contracted, and apoptosis was induced in early and late colorectal cancer cells. The expression of Caspase-9 and Fas death receptor increased, however, CYP1A1 and GSTP1, Bcl-2 decreased in the same trend in both cells; while the mRNA expression of Bax and multidrug resistance protein-2 (MRP-2) increased when BV treated HCT-116 cells and decreased when BV treated SW-480 cells. After MEL treatment, the expression of Bax decreased and MRP-2 increased in HCT-116 cells, while the expression of Bax and MRP-2 decreased in SW-480 cells (Nikodijevic et al., 2021). Besides, the high concentration of MEL could directly and quickly cause membrane damage, content outflow and cell death to gastric cancer and colorectal cancer cell membranes within 15 min. This rapid dissolution effect appeared in AGS cells, COLO205 and HCT-15 cells in different ways (Soliman et al., 2019). In addition, MEL and bvPLA_2_ inhibited HCT116 cell proliferation in a synergistic manner, demonstrating synergistic utility: MEL promoted the effect of bvPLA_2_ on cell membranes, and pretreatment of cells with bvPLA_2_ enhanced the inhibitory effect of MEL on cells (Yaacoub et al., 2021). Besides, the expression and activity of 15-lipoxygenase-1, a tumor suppressor in HT-29 cells, have elevated after being affected by BV, which in turn promoted apoptosis (Zare et al., 2019).

Meanwhile, the derivation of the side chain of oligopeptide-alginate NPs provided the basis for the specific binding of MEL, ultimately achieving potent killing ability on human cloned colon adenocarcinoma Caco-2 cells (Wattanakul et al., 2019).

### Anti-liver cancer research

In the study of hepatocellular carcinoma, BV achieved the same breakthrough as MEL in anti-hepatocellular carcinoma growth with autophagy, which implied a possible anti-mutagenic effect on normal cells. The results showed that MEL down-regulated Bcl-2 and up-regulated cytochrome C (Cyt C), Caspase-3, and Caspase-9 expression, predicting that MEL may rely on the mitochondrial apoptotic pathway to induce tumor injury, and the ratio of apoptosis to necrosis in cancer cells was positively relative to the MEL concentration. On the other hand, MEL achieved its autophagy-inhibiting effect on HepG2 cells by downregulating p62 and upregulating Beclin 1 and LC3 expression. The anti-tumor effect of MEL was enhanced when the autophagy inhibitor chloroquine was applied; the enhanced autophagic effect of MEL on hepatocellular carcinoma cells was diminished after the application of the autophagy activator rapamycin (Lv et al., 2019). The shaping of hypoxic environment is strongly associated with tumor proliferation or angiogenesis, and the vasculogenic mimicry (VM) produced by SMMC-7721 cells induced by cobalt chloride (CoCl_2_) with EMT can also be inhibited by applying MEL. The hypoxia model caused upregulation of the expression of HIF-1α, VEGF, MMP-2 and MMP-9 in SMMC-7721, Huh7, and HepG2 cells, and MEL reversed this trend. In addition, in the presence of MEL, it decreased SMMC-7721 cell viability, inhibited EMT induced by CoCl_2_, upregulated E-cadherin, and downregulated p-Akt, vimentin and N-cadherin expression. An *in vivo* tumor treatment model of MEL was established by s.c. injecting SMMC-7721 cells into male BALB/c nude mice, which showed the significantly inhibited HIF-1α expression and tumor growth (Chen et al., 2019).

Sorafenib had unsatisfactory effects in the treatment of advanced hepatocellular carcinoma, while BV and MEL had certain efficacy in inhibiting hepatocellular carcinoma. Therefore, BV and MEL alone or in combination with sorafenib, respectively, showed synergistic effects in adjuvant inhibition of HepG2 cell proliferation. The expression of p53, Bax, Caspase-3, Caspase-7 and PTEN was elevated, meanwhile the expression of Bcl-2, Cyclin D1, HIF-1α, VEGF, Ras-related C3 botulinum toxin substrate 1 (Rac1), MMP-9 and NF-κB decreased in HepG2 cells. The promotion or suppression effects on the above genes were strengthened under the crosstalk conditions (Mansour et al., 2021).

### Anti-bladder cancer and prostate cancer research

In an investigation of the Gene Expression Omnibus database of bladder cancer, MEL regulated and inhibited the expression of key module genes in the PI3K-Akt and TNF signaling pathways, referring to LPAR1, COL5A1, COL6A2, CXCL1, CXCL2 and CXCL3 in human bladder cancer cell lines T24 and 5637, and suppressed cell proliferation and migration activities, revealing the potential role of these genes as targets of MEL in bladder cancer (Jin et al., 2018). Similarly, bearing in mind the bioinformatics analysis of bladder cancer, the genes corresponding to two bladder cancer cells, UM-UC-3 and 5637, were selected for study. All these demonstrated that MEL could inhibit cell proliferation, migration and invasion by virtue of its effect on MAPK signaling pathway or V-ATPase (Yao et al., 2020).

Prostate cancer is divided into metastatic/non metastatic prostate cancer. As one of the familiar type diseases in masculinity, it faces several problems with drug resistance of cancer cells and inability to control the progress and spread of the disease. BV and MEL have certain effects on a variety of prostate cancer and xenotransplantation (Badawi, 2021). For example, BV produced selective antitumor effects on PC3 cells, reducing their cell viability (Viana et al., 2021).

### Anti-skin cancer research

Melanoma stands out as one of the most lethal and invasive malignancies in skin cancer, yet it is highly resistant to drugs. BV and MEL can help to fight against the growth, migration and invasion of melanoma A375SM, B16F10 and SK-MEL-28 cells, causing apoptosis. Among them, MEL showed a more effective ability to inhibit migration and promote apoptosis. BV and MEL had similar inhibitory effects on PI3K/Akt/mTOR and MAPK signaling pathways in A375SM cells. At the same time, it elevated the cleaved Caspase-3 and Caspase-9 expression and reduced the microphthalmia-associated transcription factor (MITF) level. In addition, when MEL was combined with temozolomide, the growth and invasion inhibition of A375SM and SK-MEL-28 cells elevated (Lim et al., 2019). MEL from *Apis florea* (MEL-AF) similarly showed a proliferation inhibitory effect on A375 cells, where MEL-AF, upon binding to the cell membrane, caused an elevation of intracellular F-actin with a decrease in EGFR, ultimately resulting in apoptosis through the induced expression of Cyt C, Caspase-3 and Caspase-9 in the mitochondrial apoptotic pathway (Sangboonruang et al., 2020).

MEL alone or in combination with 5-fluorouracil was able to damage A431 cells of skin squamous cell carcinomas, causing morphological alternations, e.g., cell shedding, shrinkage, and plasma membrane damage. Besides, the combination of MEL and 5-fluorouracil caused a more significant decrease in terms of cell number and cell cycle arrest in both phases S and G2/M. More importantly, the drug combination re-sensitized A431 cells to 5-fluorouracil (Ombredane et al., 2021).

Head and neck squamous cell carcinoma (HNSCC) is also a type of skin cancer. Four types of HNSCC cells viability such as UMSCC12, UMSCC29, UMSCC38 and UMSCC47 were inhibited by BV alone or combined with cisplatin. Besides, mitosis was blocked in G2/M phase, during which the Bcl-2 and EGFR expression was significantly reduced, while the expression of Bax was significantly elevated. It is worth noting that different drugs and ratios of treatment were shown to significantly reduce the number of the S-phase cells (Grawish et al., 2020).

### Anti-leukemia research

MEL induced apoptosis while inhibiting cell viability in CCRF-CEM and K562 cells, relying on activation of the hydrolytic activity of Caspase-3/7 in the mitochondrial pathway and the hemiphilic aspartate pathway (Ceremuga et al., 2020). In addition to inhibiting cell viability, MEL had high permeability to the plasma membrane of cells in human acute T cell leukemia Jurkat cells, which enhanced the permeability of MEL through the plasma membrane and further caused cell death (Gasanoff et al., 2021). The anti-infectivity peptide melectin from *Melecta albifrons*, by virtue of its α-helical structure, inhibited cell proliferation by interfering with the cell membrane of leukemic cells K562, decreasing the viability of various cells such as K562, K562/ADM and HL-60 while enhancing LDH output (Liang et al., 2021).

### Anti-other cancer research

In a study of Hodgkin lymphoma, MEL produced toxicity in lymphoma cells L-428 and KM-H2, while increasing the sensitivity of drug-resistant L-428 cells to cisplatin. And MEL preferentially acted on tumor cells, demonstrating prospect of Hodgkin lymphoma therapy in the future (Kreinest et al., 2021). Besides, Cyclin D, MMP-2, MMP-9, lipoprotein receptor related protein 5 (LRP5), β-catenin and other proteins associated with the Wnt/β-catenin pathway were downregulated after moderate and high concentrations of MEL on human osteosarcoma 143B cells, a malignant bone tumor. The s.c. injection of 143B cells and treatment with MEL in female BALB/c^nu/nu^ nude mice showed a reduction in tumor size, mass and number of lung metastatic nodules, and inhibition of tumor metastatic behavior (Zhu et al., 2021). Glioblastoma multiforme is also a malignant tumor. BV and MEL reduced the viability of Hs683, T98G and U373 cells, elevated Bak and Bax expression, inhibited Caspase-3 expression as well as promoted late apoptosis and necrosis in glioblastoma multiforme. In addition, the expression of long-chain non-coding RNARP11-838N2.4 and X inactive-specific transcript (XIST) was significantly elevated in glioblastoma multiforme cells (Lebel et al., 2021). BV or MEL alone inhibited the growth of Ehrlich ascites carcinoma cells. Injection of BV or MEL into female albino tumor-bearing mice resulted in destruction of tumor tissue and suppression of tumor size. In addition, after combined treatment with γ-radiation, the tumor size inhibition was enhanced by re-enforcing the elevated levels of TNF-α, VEGF-A, serum MMP-2 and MMP-9, and CAT in liver caused by BV or MEL alone (El Bakary et al., 2020).

Lipodisk-based paclitaxel and MEL co-delivery system functionalized with glycopeptide ^9^G-A7R (^9^G-A7R-Disk/PTX/MEL) were used as an anti-degradation delivery system for MEL on U87 glioma cells cultured *in vitro* contributing to the growth inhibitory effect. Besides, inoculation of U87 cells in female BALB/c nude mice and intravenous (i.v.) administration of ^9^G-A7R-Disk/PTX/MEL co-loaded liposomes resulted in increased apoptosis, tissue damage, and reduced angiogenesis at the glioma, demonstrating their targeted anti-tumor effects (Wang et al., 2019).

## Effects on neurological disorders

**FIGURE 3:**
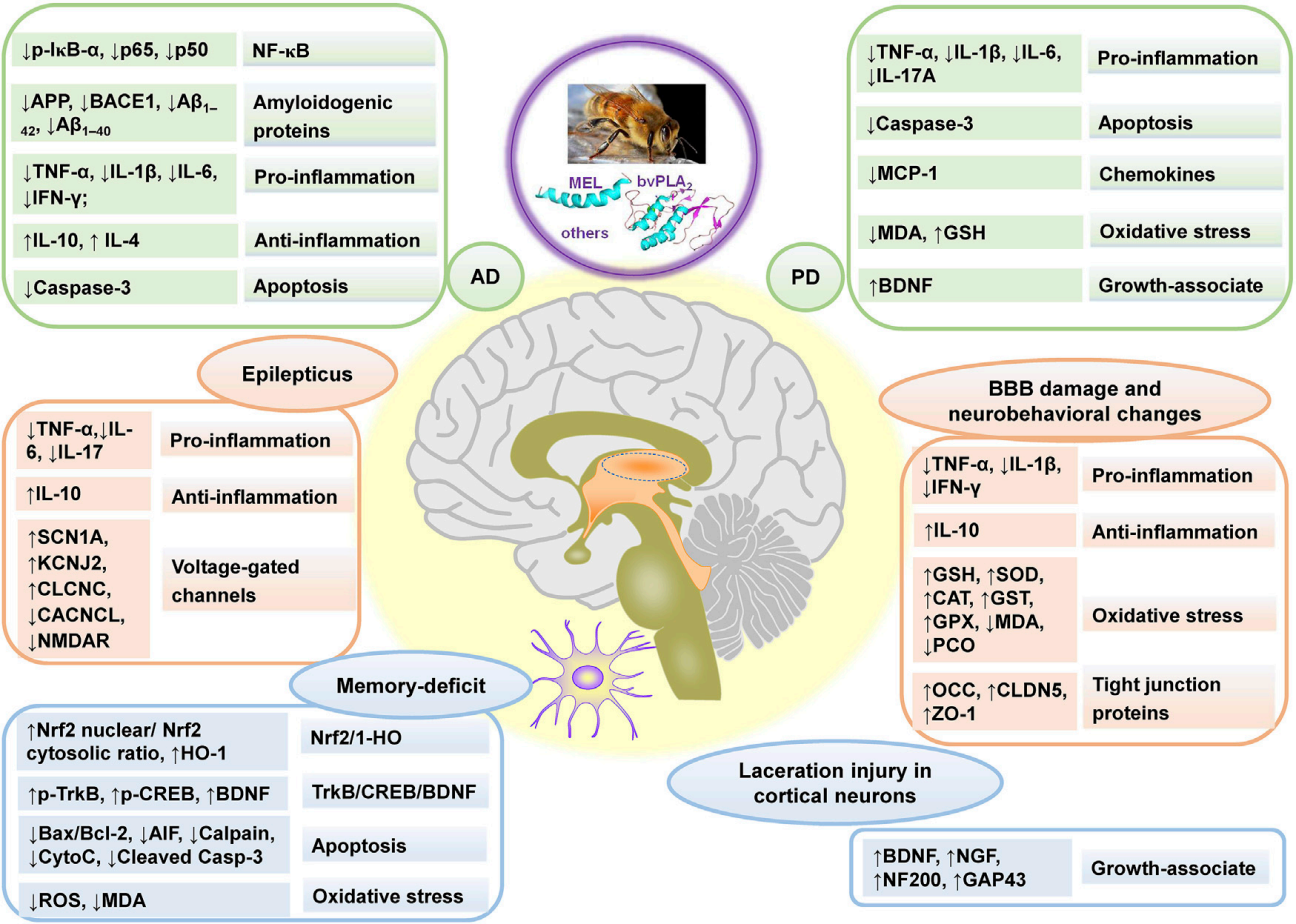
The main affected targets and mechanism of BV and its main components in treating neurological disorders, referring to Alzheimer’s disease (AD), Parkinson’s disease (PD), BBB damage and neurobehavioral changes, laceration injury in cortical neurons, memory-deficit, and epilepticus. “↑” and “↓” represent up-regulated and down-regulated targets (genes or proteins), respectively in the left column in each rounded rectangular box, and the texts demonstrate the effect and pathways in right column in each rounded rectangular box for BV and its main components.

Parkinson’s disease (PD) and Alzheimer’s disease (AD), belonging to neurodegenerative diseases, are caused by nervous system abnormalities, involving neurotransmitter abnormalities, the accumulation of false proteins, etc. (Guo and Ma, 2019). BV and its main component, bvPLA_2_, showed neuroprotective effects and could postpone the progression of degenerative diseases. The effects mainly included enhancing motor performance or alleviating memory impairments, inhibiting oxidative stress, decreasing neuroinflammation, protecting neurons, preventing apoptosis, etc. Besides, BV and its main components also had neuroprotective effects against other neurological disorders, seen in Table 2, and the main affected targets and mechanism of BV and its main components in treating neurological disorders is shown in Figure 3.

**TABLE 2:** Summary of the role and mechanism of BV and its main components in treating neurological disorders.

BV and/or its components	Model	Inducer/Method	Animal/Cell (BV etc. administration)	Effects	Mechanisms	References
PD						
BV	PD	Rotenone (s.c.)	Swiss albino male mice (0.065, 0.13, and 0.26 mg/kg, intradermal (i.d.), once a day every other day for 2 weeks)	Enhance motor performance; inhibit oxidative/nitrosative stress; decrease neuroinflammation; protect dopaminergic neurons	↓MDA, ↓NO, ↑GSH, ↑PON1 activity, ↑TAC; ↓MCP-1, ↓TNF-α, ↓Caspase-3, ↑BuChE activity, ↑DA	Badawi et al. (2020)
BV	PD	Rotenone (s.c.)	Swiss Albino male mice (1.0 mg/kg, i.p., for 6 consecutive days)	Recover motor strength and motor coordination; increase dopamine level and total antioxidant capacity; reinforce anti-inflammatory effect; restrict neuronal degeneration	↑DA, ↓IL-1β, ↓IL-6	Rakha et al. (2018)
BV	PD	Reserpine (i.p.)	Male rats (10 μL/kg, i.p., every other day for 30 days)	Increase monoamines neurotransmitters (norepinephrine, dopamine, 5-HT), elevate γ-aminobutyric acid and arginine, reduce glutamate, halt DNA fragmentation	↓acetylcholinesterase activity, ↓TNF-α, ↓IL-1β, ↑PON1 activity, ↑BDNF	Ahmed-Farid et al. (2021)
bvPLA_2_	PD	1-Methyl-4-phenyl-1,2,3,6-tetrahydropyridine (MPTP) (i.p.)	C57BL/6J mice (0.5 mg/kg, s.c., for a consecutive 6 days)	Improve motor function; rescue loss of dopaminergic neurons	Activate Tregs; inhibit Th1 and Th17 cells	Kyung Hwa Kim et al. (2019a)
bvPLA_2_	PD	MPTP (i.p.)	Male C57BL/6J mice (0.5 mg/kg, i.p., s.c., i.m., or i.v., for 6 days; 0.01–0.5 mg/kg, s.c., for six consecutive days)	Reverse motor deficits; inhibit loss of dopaminergic neurons; suppress microglial activation (↓Iba1-positive microglia, ED1^+^ microglia)	Induce CD4^+^CD25^+^Foxp3^+^ Tregs; inhibit Th1 and Th17 polarization (↓IFN-γ, ↓IL-17A)	Baek et al. (2018a), Kyung Hwa Kim et al. (2019b)
Fraternine (a novel Wasp peptide)	PD	6-Hydroxydopamine (Nigro-striatal pathway lesioned unilaterally)	Swiss male mice (0.01, 0.1, 1 and 10 µg/animal, i.c.v, on day 1, 3 and 5)	Trigger neuroprotective activity; improve motor coordination	—	Biolchi et al. (2020)
AD						
BV	AD	Aβ	U87MG (human glioblastoma cells) (10 μg/ml, for 120 h)	Increase cell viability; decrease Aβ accumulation; suppress inflammatory reaction; prevent apoptosis	↓COX-2, ↓TNF-α, ↓IL-1, ↓Caspase-3	Ku et al. (2020)
bvPLA_2_	AD	Aβ1-42 peptide (s.c.)	AD model (3xTg-AD) mice (0.5 mg/kg, i.p., once a week for 3 months)	Alleviate memory impairments; reduce Aβ burdens in the hippocampal CA1 and cortex regions; high cerebral glucose uptake; eliminate central nervous system inflammation	↓IFN-γ, ↓TNF-α, ↑IL-10	Baek et al. (2018b)
		Aβ1-42 peptide (s.c.) and pertussis toxin (PT) (i.v.)	Male C57BL/6 mice (0.5 mg/kg, i.p., once a week for 3 months)	Eliminate central nervous system inflammation	—	
BV sPLA_2_	AD	LPS (i.p.)	Male ICR mice (0.02, 0.2, and 2 mg/kg, i.p., three times for 1 week)	Improve memory function; inhibit Aβ deposition; inhibit neuroinflammation and NF-κB activation; modulate Tregs infiltration	↓GFAP, ↓IBA-1, ↓iNOS, ↓COX-2, ↓p-IκB-α, ↓p50, ↓p65, ↑Foxp3	Ham et al. (2019a)
		LPS	Microglial BV-2 cells (0.01, 0.1, and 1 μg/ml, for 3 h)	Reduce amyloidogenesis and neuroinflammation	↓APP, ↓BACE1, ↓β-secretase activity, ↓iNOS, ↓COX-2, ↓IBA-1, ↓p-IκB-α, ↓p50, ↓p65, ↓TNF-α, ↓IL-1β, ↓IL-6	
bvPLA_2_	AD	—	Tg2576 mice (1 mg/kg, i.p., twice per week for 4 weeks)	Alleviate genetically induced memory; inhibit accumulation of Aβ	↓APP, ↓BACE1, ↓Aβ_1–42_, ↓Aβ_1–40_, ↓β-secretase activity, ↓GFAP, ↓IBA-1, ↓iNOS, ↓COX-2, ↓TNF-α, ↓IL-1β, ↓IL-6, ↑IL-4, ↑TGF-β, ↓p-STAT3, ↓p-ERK	Ham et al. (2019b)
		LPS	BV-2 cells (0.01, 0.1, and 1 μg/ml, for 24 h)	Inhibit accumulation of Aβ, decrease nitric oxide concentration; directly binds to linker domain of STAT3	↓APP, ↓BACE1, ↓Aβ_1–42_, ↓β-secretase, ↓iNOS, ↓COX-2, ↓IBA-1, ↓TNF-α, ↓IL-1β, ↓IL-6, ↑IL-4, ↑TGF-β, ↓p-STAT3	
		Aβ	BV-2 cells (0.01, 0.1, and 1 μg/ml, for 24 h)	—	↓iNOS, ↓COX-2, ↓p-STAT3, ↓TNF-α, ↓IL-1β, ↓IL-6	
Other neurological disorders						
BV	Epilepticus	Pilocarpine	Male Sprague Dawley rats (10 µg/animal, i.d., once every 3 days for four consecutive weeks)	Ameliorate disturbance of electrolytes and the interruption of electrolytes and ions, limit neuronal excitability via rapid repolarization of action potentials	↑SCN1A, ↑KCNJ2, ↑CLCNC, ↓CACNCL, ↓NMDAR, ↓IL-6, ↓IL-17, ↓TNF-α, ↓TGF-β, ↓FOXP3, ↓CTL4, ↑IL-10	Abd El-Hameed et al. (2021)
BV	Blood brain barrier damage and neurobehavioral changes	Methyl mercury chloride (gavaged)	Male Sprague–Dawley rats (0.5 mg/kg, s.c., for14 days)	Modulate methyl mercury chloride-induced behavioral alterations, increase pan neuron	↑GSH, ↑SOD, ↑CAT, ↑GST, ↑GPx, ↓MDA, ↓PCO, ↓8-hydroxy-2′-deoxyguanosine, ↑IL-10, ↓NO, ↓TNF-α, ↓IL-1β, ↓INF-γ, ↑occludin, ↑claudins-5, ↑Zonula occludens-1, ↓TGF-β, ↓IgG, ↓IBA-1	Abu-Zeid et al. (2021)
MEL	Memory-deficit	Aβ_25–35_	HT22 mouse hippocampal cell (0.3, 1, and 3 μM, for 24 h)	Reduce apoptosis, decrease protein carbonyl levels	↓Bax/Bcl-2, ↓AIF, ↓Calpain, ↓Cyt C, ↓cleaved Caspase-3, ↓ROS, ↓MDA, ↓LDH, ↑Nrf2 nuclear/Nrf2 cytosolic ratio, ↑HO-1, ↑p-TrkB, ↑p-CREB, ↑BDNF	Cong Duc and Lee, (2021)
		Aβ_25–35_ (intracerebroventricular)	Male ICR mice (0.15 and 1.5 mg/kg, s.c., on days 3, 5, 7, 9, and 11)	Improve memory impairment, increase neuron cell neurogenesis; reduce acetylcholinesterase activity, increase acetylcholine	↓ROS, ↓NO, ↓MDA, ↑p-CREB, ↑BDNF, ↓iNOS, ↑M1 muscarinic acetylcholine receptor	
Apamin	Laceration injury in cortical neurons	Laceration injury	Rat cerebral cortical neurons (1 and 10 μg/ml, for 24 and 48 h)	Enhance neurite outgrowth and axon regeneration, increase BDNF-positive and NGF-positive neurons	↑BDNF, ↑NGF	Aeyung Kim et al. (2021)
		—	Rat cerebral cortical neurons (1 and 10 μg/ml, for 24 and 48 h)	—	↑BDNF, ↑NGF, ↑NF200, ↑GAP43	
BV, Wasp venom	Neuroinflammation	LPS	BV-2 murine microglial cells (0.5, 1, 2, and 4 μg/ml (BV), 5, 10, 20, and 40 μg/ml (Wasp venom), for 18 or 24 h)	Anti-inflammatory effect	↓NO, ↓TNF-α, ↓IL-6, ↓iNOS, ↓COX-2, ↓NF-κB	Yun et al. (2021)
Apamin	Neuroinflammation	LPS	BV-2 murine microglia cells (1 μg/ml, for 1 h)	Anti-neuroinflammatory effect	↓CD11b, ↓TNF-α, ↓IL1β, ↓IL6, ↓COX2, ↓K_Ca_2.2, ↓pCaMKII, ↓TLR4, ↓pp65, ↓pSTAT3, ↓pERK, ↓pJNK	Park et al. (2020)
		LPS	Rat primary microglial cells (1 μg/ml, for 1 h)	Anti-neuroinflammatory effect	↓TNF-α, ↓IL1β, ↓CD11b, ↓K_Ca_2.2, ↓pCaMKII, ↓TLR4, ↓pp65, ↓pSTAT3, ↓pERK	
Apamin	Multiple sclerosis	Cuprizone pellets (fed)	Male C57BL/6 mice (100 μg/kg/BW, i.p., during phase I (demyelination) or post-treatment phase II (remyelination) twice a week)	Increase Olig2+cells in phase I, show a decreasing trend in PDGFRa + cells after cuprizone withdrawal; stimulate oligodendrocyte progenitor cell proliferation in phase I, especially at the subventricular zone	—	Danesh-Seta et al. (2021)
bvPLA_2_	Multiple sclerosis (experimental autoimmune encephalomyelitis)	MOG_35-55_ peptide in CFA containing *Mycobacterium tuberculosis* (s.c.) and pertussis toxin (i.p.)	C57BL/6 mice (0.2 mg/kg, i.p., daily for a period of 10 days)	Attenuate limb paralysis, decrease CD4^+^ cell infiltration; the beneficial effects of bvPLA_2_ disappeared when Tregs were depleted	—	Gihyun Lee et al. (2019)

Abbreviations are as shown in the literature. (*↓*), down-regulation or inhibition; (*↑*), up-regulation or activation.

### Delaying the development of PD

BV was reported to have neuroprotective effect on dopaminergic neurons and alleviate PD symptoms. BV attenuated motor impairment, decreased oxidative/nitrosative stress, and TNF-α, Caspase-3, and monocyte chemoattractant protein-1 (MCP-1) expression, and increased dopamine (DA) content and butyrylcholinesterase (BuChE) activity in a rotenone-induced PD mice model (Badawi et al., 2020). Besides, BV restored the levels of DA, norepinephrine and serotonin (5-HT), balanced glutamate/γ-aminobutyric acid levels, prevented DNA fragmentation, reduced TNF-α and interleukin-1β (IL-1β), and increased the brain-derived neurotrophic factor (BDNF) and paraoxonase 1 (PON1) level in a reserpine-induced PD rat model (Ahmed-Farid et al., 2021). The above results implied that BV could be a potential adjuvant for PD treatment. The neuroprotective effects of bvPLA_2_ against PD have also been studied. Purified bvPLA_2_ showed dose-dependent neuroprotective effects on PD in mice, relating to the induction of CD4^+^CD25^+^Foxp3^+^ regulatory T cells (Tregs), which to some extent suppressed the polarization of T helper 1 (Th1) and Th17, and the microglia activation (Kim et al., 2019b). Fraternine, a novel wasp peptide, also showed neuroprotective effects and ameliorated motor coordination in a 6-hydroxydopamine-induced PD mice model (Biolchi et al., 2020).

Besides, current evidence was summarized and supported the therapeutic effects of acupuncture in treating PD patients and animal models of PD (Guo and Ma, 2019). Therefore, BV combined with acupuncture could have great advantages in the treatment of PD.

### Delaying the development of AD

BV increased cell viability, decreased amyloid β-protein (Aβ) accumulation in U87MG AD mimic cells, as well as suppressed inflammatory reaction through inhibiting the mRNA expression of IL-1, TNF-α and cyclooxygenase-2 (COX-2), and prevented apoptosis by reducing the expression level of Caspase-3, indicating that BV could be a potential AD therapeutic drug (Ku et al., 2020). Besides, bvPLA_2_ also exerted neuroprotective effects against AD. It alleviated memory impairments, reduced Aβ burdens, showed high cerebral glucose uptake, and eliminate central nervous system inflammation through reducing TNF-α and interferon-γ (IFN-γ) level and elevating IL-10 level in a 3xTg-AD mouse model (Baek et al., 2018b). BV sPLA_2_ improved memory function, suppressed Aβ deposition, inhibited neuroinflammation and NF-κB activation through the downregulation of glial fibrillary acidic protein (GFAP), ionised calcium binding adaptor molecule 1 (IBA-1), inducible nitric oxide synthase (iNOS), COX-2, p-IκB-α, p50 and p65, and modulated Tregs infiltration through the upregulation of Foxp3 in a lipopolysaccharide (LPS)-induced AD mouse model brain. Besides, it reduced amyloidogenesis and neuroinflammation by reducing the level of amyloid precursor protein (APP), β-amyloid precursor protein-cleaving enzyme-1 (BACE1), iNOS, COX-2, IBA-1, p-IκB-α, p50, p65, TNF-α, IL-6, IL-1β, and the activity of β-secretase in LPS-treated microglial BV-2 cells. The *in vivo* and *in vitro* results indicated that BV sPLA_2_ inhibited inflammatory responses and amyloidogenesis via blockage of NF-κB signaling (Ham et al., 2019a). In addition, bvPLA_2_ also exerted anti-inflammatory and anti-amyloidogenic effects via inhibiting signal transducer and activator of transcription 3 (STAT3) activity (Ham et al., 2019b).

### Effects on other neurological disorders

BV and its main components, such as MEL and apamin, also had neuroprotective effects against other neurological disorders, including epilepticus, blood brain barrier damage and neurobehavioral changes, memory-deficit, laceration injury in cortical neurons, neuroinflammation and multiple sclerosis.

BV rebalanced neurotransmitters and blood electrolytes, ameliorated alterations of voltage-gated channels expression, and regulated pro- and anti-inflammatory cytokines levels in a pilocarpine-induced epilepticus rat model, which demonstrated that BV could slow down the development of epilepticus as a combined treatment with other antiepileptic drugs (Abd El-Hameed et al., 2021). Besides, Egyptian BV ameliorated blood-brain barrier dysfunction and neurobehavioral toxicity in rats induced by methyl mercury chloride through regulation of the methyl mercury chloride altered behavioral responses, gene expression of tight junction proteins, and immune-expression markers for specific neural cell types (Abu-Zeid et al., 2021).

MEL exerted neuroprotective effects on HT22 cells treated with Aβ_25–35_ through activation of nuclear factor (erythroid-derived 2)-like 2 (Nrf2)/heme oxygenase-1 (HO-1), and tropomyosin-related kinase receptor B (TrkB)/cAMP response element-binding (CREB)/BDNF signaling pathways. Additionally, MEL restored exhausted learning and memory abilities in an Aβ_25–35_-induced cognitive deficits mouse model. The above results showed that MEL could be a candidate agent for neurodegenerative disorders (Cong Duc and Lee, 2021). Apamin enhanced neurite outgrowth and axon regeneration after laceration injury, and increased the expression of BDNF, nerve growth factor (NGF) and regeneration-related genes in mature cortical neurons (Kim H. et al., 2021). Apamin inhibited LPS-induced neuroinflammatory responses in BV2 and rat primary microglial cells. It significantly inhibited proinflammatory cytokine production and microglial cell activation by downregulating the expression of pCaMKII and toll-like receptor 4 (TLR4) (Park et al., 2020). Especially, apamin inhibited the translocation of p65/STAT3 and MAPK-ERK signaling, which was verified through inhibitors (Park et al., 2020). The above findings suggested that apamin could be a potential adjuvant for treating a variety of neurological diseases. Besides, Apamin and bvPLA_2_ contributed to the control of multiple sclerosis (Lee G. et al., 2019; Danesh-Seta et al., 2021).

## Alleviating inflammatory diseases

**FIGURE 4:**
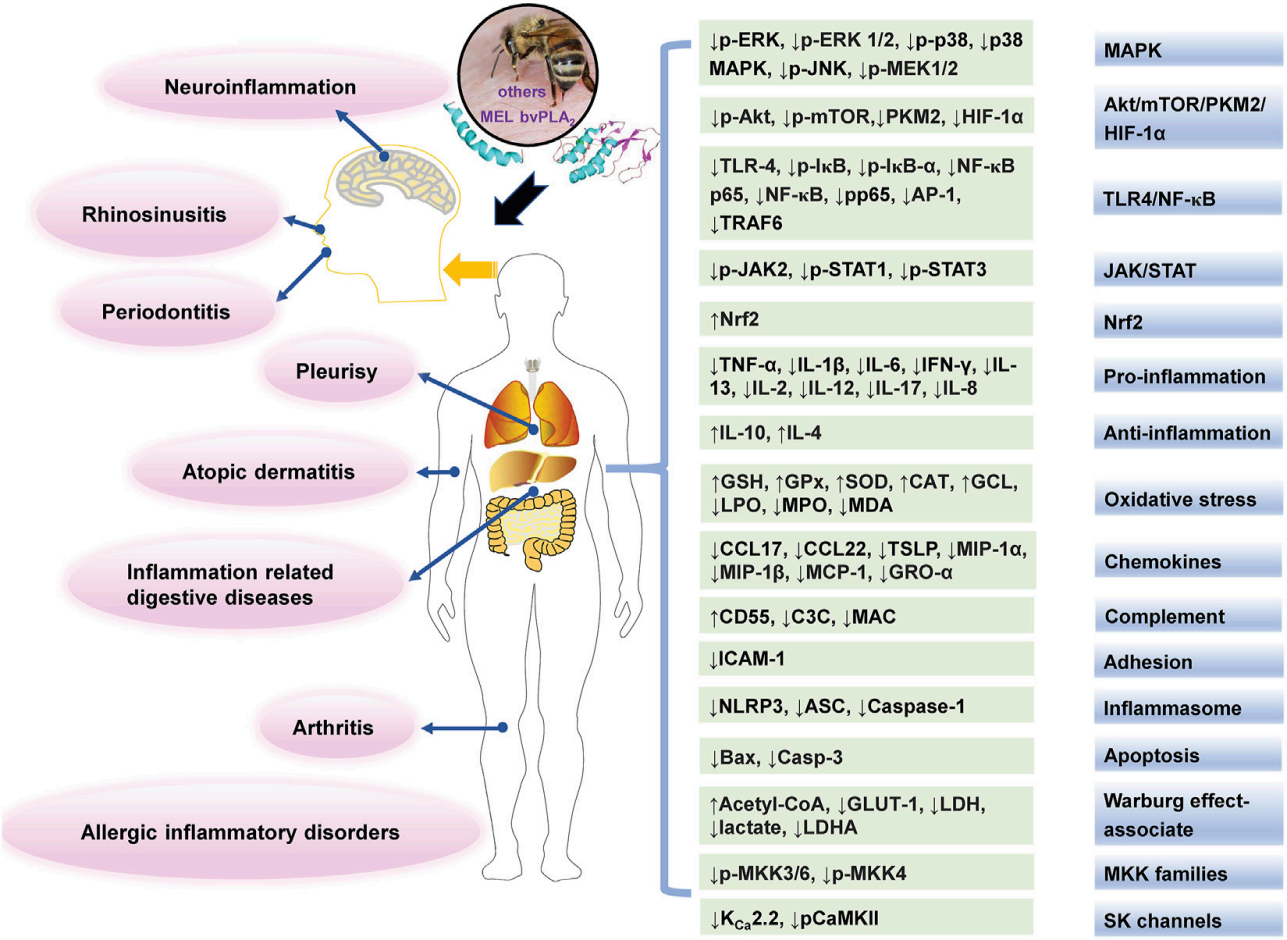
The main affected targets and mechanisms of BV and its main components in alleviating inflammatory diseases. The texts in pink oval boxes, light grey green boxes, and gradient grey blue boxes indicate related diseases, regulated targets and pathways (or mechanisms), respectively.

Chronic inflammation could cause the development of many diseases, such as skin diseases and RA (Wehbe et al., 2019). In classical medicine, BV and its main components were used for treating chronic inflammatory disorders. Recent studies are shown in Table 3, and the main affected targets and mechanism of BV and its main components in alleviating inflammatory diseases is shown in Figure 4.

**TABLE 3:** Summary of the effects and mechanisms of BV and its main components on inflammatory diseases.

BV and/or its components	Model	Inducer/Method	Animal/Cell (BV etc. administration)	Effects	Mechanisms	References
Atopic dermatitis						
BV	Atopic dermatitis	Chicken ovalbumin (i.p. inoculated)	Female BALB/c mice (1, 10, and 100 μg/kg, i.p., twice a week)	Decrease the extent of inflammatory cell infiltration and skin thickness, diminish the extent of mast cell infiltration and degranulation, increase filaggrin	↓IgE, ↓TNF-α, ↓TSLP, ↓CD4+, ↓CD11b+	Gu et al. (2018)
BV	Atopic dermatitis	Phthalic anhydride (topical application)	BALB/c mice (200 µL of a solution containing 0.1, 0.25, and 0.5 μg, topical application, 3 h)	Reduce atopic dermatitis clinical score, back and ear epidermal thickness, the weight of lymph node; decrease the number of eosinophil, neutrophil, monocytes, mast cells, F4/80-positive cells and Ly6G-positive cells	↓IgE, ↓IL-4, ↓IL-13, ↓TNF-α, ↓IL-1β, ↓IL-6, ↓p-ERK, ↓p-p38, ↓p-JNK, ↓p-IκB-α, ↓iNOS, ↓COX-2	Ji Hwan Lee et al. (2020)
		LPS	RAW 264.7 cells (1, 2.5, and 5 μg/ml, for 24 h)	Anti-inflammatory effect	↓NO, ↓NF-κB, ↓p-ERK, ↓p-p38, ↓p-JNK, ↓p-IκB-α, ↓iNOS, ↓COX-2, ↓p65, ↓p50	
		TNF-α/IFN-γ	HaCaT cells (1, 2.5, and 5 μg/ml, for 24 h)	Anti-inflammatory effect	↓NF-κB, ↓p-ERK, ↓p-p38, ↓p-JNK, ↓p-IκB-α, ↓p65, ↓p50	
BV and MEL	Atopic dermatitis	DNCB (topically to the shaved dorsal skin)	Female Balb/c mice (100, 200 and 500 μg, topical application, five times per week for 4 weeks)	Decrease dorsal skin thickness; inhibit pathological changes including the infiltration of inflammatory cells in skin lesions; decrease the levels of CD4^+^ and CD3^+^ T cells in the dorsal skin; improve abnormal epidermal differentiation	↓IFN-γ, ↓IL-4, ↓IgE, ↓TSLP	An et al. (2018)
		TNF-α/IFN-γ	Human keratinocyte HaCaT cell line (1, 10 and 100 ng/ml (BV), 0.1, 0.5 and 1 μg/ml (MEL), for 9 h)	Modulate chemokines expression via suppression of pro-inflammatory cytokines; inhibit JAK/STAT signal pathways; inhibit NF-κB pathways	↓CCL17, ↓CCL22, ↓IL-1β, ↓IL-6, ↓IFN-γ, ↓p-JAK2, ↓p-STAT1, ↓p-STAT3, ↓p-IκB, ↓p-NF-κB	
BV	Atopic dermatitis (Irritant contact dermatitis, ICD)	DNCB (topical application)	Male BALB/c mice (0.3 mg/kg, s.c., at 2-day intervals for 10 days)	Attenuate skin condition, decrease clinical skin score; inactivate complement system	↓C3C, ↓MAC, ↑CD55 (BV and MEL)	Yenny Kim et al. (2019)
		—	THP-1 cells (0–1 μg/ml, for 0–24 h or 0–120 min)	Inactivate complement system	↑CD55, ↑p-ERK1/2	
bvPLA_2_	Atopic dermatitis	DNCB and house dust mite extract (*D. farinae* extract) (topically to the ear)	Male C57BL/6 mice (80 ng/ear, topical application, four times a week for 3 weeks)	Suppress atopic dermatitis-related skin swelling, improve ear thickness; decrease the expression of Th1 and Th2 cytokines; induce Treg; decrease epidermal and dermal thickness and macrophage infiltration; block mast cell infiltration	↓IgE, ↓IFN-γ, ↓IL-4, ↓IL-6, ↓IL-10, ↑Foxp3	Dasom Shin et al. (2018)
BV	Atopic dermatitis	House dust mite (*D. farinae*) extract	HaCaT cells (0.1–10 μg/ml, for 24 h)	Anti-inflammatory effects	↓protease-activated receptor 2 (PAR2), ↓intercellular adhesion molecule-1 (ICAM-1), ↓IL-6	Han et al. (2018)
Arthritis						
BV	RA	Freund’s complete adjuvant (sub-plantar, intra-dermally)	Male Wistar Albino rats (2, 4 and 20 mg/kg, s.c., every day for a period of 15 days)	Decrease paw volume and arthritis index; prevent DNA damage	↓total oxidant status, ↓oxidative stress index, ↑total antioxidant status, ↓MPO, ↓IL-1β, ↓IL-6, ↓TNF-α, ↓TGF-β1, ↓RF, ↓CRP, ↓ASO	Kocyigit et al. (2019)
BV and hesperidin	RA	Complete Freund’s adjuvant (s.c., right hind paw)	Male Wistar rats (1 mg/kg b.w., s.c. (BV), 25 mg/kg b.w, oral gavage (hesperidin), daily for 3 weeks)	Decrease paw edema, the leukocytosis, lymphocyte, monocyte, neutrophil and eosinophil counts; counteract severe inflammatory changes and leukocytic infiltration in the periarticular tissue of the ankle joints (BV and/or hesperidin); amend the lymphoid hyperplasia in white pulps of spleen and the widening of the medulla and mononuclear cell infiltration found in thymus (BV and hesperidin)	↑GSH, ↑GPx, ↓IL-2, ↓IL-12, ↓TNF-α (BV); ↓LPO, ↑GSH, ↑SOD, ↑GPx, ↑IL-10, ↓IL-2, ↓IL-12, ↓TNF-α, ↑IL-4 (BV and hesperidin)	Ahmed et al. (2018)
Wasp venom	RA	—	MH7A cells (0, 12.5, 25, 50, and 100 μg/ml)	Inhibit viability, induce MH7A synovial cell apoptosis	↑Caspase-3, ↑Bax, ↓Bcl-2	Liu et al. (2021)
bvPLA_2_	RA	Collagen-induced arthritis and incomplete Freund’s adjuvant (s.c.)	Male DBA/1 mice (0.1, 0.5, 1.0 mg/kg, i.p., for 5 weeks)	Inhibit body weight loss, alleviate squeaking score, paw thickness, and arthritis index; alleviate histological signs of collagen-induced abnormalities in the knee joints; anti-arthritic effects were blocked by selective Treg depletion	—	Choi et al. (2021)
Polymeric microneedle-mediated transdermal delivery of MEL	RA	Freund’s adjuvant (injected into hint foot pad)	Male SD rats (100 μg, transdermal, every other day for 8 times)	Decrease paw thickness, clinical score, suppress RA progression, preserve cartilage integrity, reduce infiltration of leukocytes and WBC level	—	Du et al. (2021)
			Male BALB/c mice (15 μg, transdermal, every other day for 6 times)	Decrease paw thickness, clinical score, reduce symptoms of RA, reduce infiltration of inflammatory cells, preserve integrity of cartilages; increase lymphocytes, decrease monocytes and granulocytes	↓TNF-α, ↓IL-17; increase CD3CD4CD25Foxp3^+^ cells	
MEL	Osteoarthritis	IL-1β	C518 cells (0, 0.1, 0.5, 1, and 10 μg/ml, for 24 h)	Inhibit NF-κB activation by preventing IκB degradation and NF-κB migration	↓iNOS, ↓NF-κB	Tang et al. (2021)
BV and apamin	Gouty arthritis	MSU (i.d. injection into the right paw)	C57BL/6 male mice (0.5 and 1 mg kg, i.p., once daily for 3 days)	Decrease paw edema, reverse the change in weight-bearing distribution; decrease MSU crystal formation	↓IL-1β, ↓IL-6, ↓TNF-α, ↓NLRP3, ↓ASC, ↓Caspase-1, ↓NF-κB	Yun Mi Lee et al. (2020)
		MSU	Murine macrophage RAW 264.7 cells (1–5 μg/ml, for 1 h)	−	↓TNF-α, ↓p-IκB-α	
BV	Gouty Arthritis	MSU (intra-articular injection)	Male SD rats (0.5 mg/kg, tibiotarsal intra-articular injection)	Mitigate paw edema and mechanical allodynia, suppress neutrophil infiltration, reduce progression of synovitis	↓MIP-1α, ↓MIP-1β, ↓MCP-1, ↓GRO-α, ↓MIP-2α, ↓iNOS, ↓COX-2	Goo et al. (2021)
Inflammation related digestive diseases						
BV	Gastric ulceration	Acetylsalicylic acid (oral administration)	Male Sprague-Dawley rats (2 mg/kg BW, i.p., for 7 days)	Gastric mucosa was nearly normal, with slight congestion; attenuate haematological, haemostatic, and histopathological alterations, reduce tissue eosinophil level; decrease ulcer index, fluid volumes, and pepsin concentrations	↓TNF-α, ↓MPO, ↓MDA, ↑SOD, ↑GSH, ↑Hsp70, ↓Bax, ↓Caspase-3	Mohamed et al. (2019)
BV	Nonalcoholic steatohepatitis	Fructose (drinking water)	Male Wistar albino rats (0.1 mg/kg, i.p., 3 times per week during the last 2 weeks)	Mitigate body weight and epididymal fat weight; increase blood glucose, decrease insulin concentration and HOMA; decrease serum and liver total lipids and TGs, total and LDL cholesterol, increase HDL cholesterol; normalize liver ODS, minute focal hepatocellular necrosis associated with inflammatory cell infiltration	↓SREBP1c, ↓SREBP2, ↑GCL, ↑Nrf2, ↓MDA, ↑intestinal tight junction proteins, ↓TNF-α, ↑LXRα, ↑FXR	Abd El-Haleim, (2020)
MEL	Ulcerative colitis	Acetic acid (intrarectal administration)	Swiss albino male mice (40 μg/kg, p.o., once per day for 5 days)	Increase body weight gain, decrease colon mass index; preserve colon mucosa and submucosa	↓TNF-α, ↓IL-6, ↓TLR4, ↓TRAF6, ↓p38 MAPK, ↓NF-κB, ↓PGE2, ↓COX-2, ↓sPLA_2_, ↓MDA, ↑SOD, ↑GSH	Ahmedy et al. (2020)
MEL	Acute liver failure	D-galactosamine/LPS) (i.p.)	Male C57BL/6 mice (2, 4, and 8 mg/kg, i.p.)	Improve survival, hepatic functions, gross liver appearance and histological changes, decrease hepatocyte death, alleviate hepatic inflammation; induce no obvious *in vivo* toxicity; repress Warburg effect	↓Total bilirubin, ↓ALT, ↓AST, ↓TNF-α, ↓IL-1β, ↓p-Akt, ↓p-mTOR, ↓PKM2, ↓HIF-1α, ↓TNF-α, ↓IL-1β	Fan et al. (2020)
		LPS	RAW264.7 murine macrophages (0.35, 0.70, 1.40, and 2.80 μM, for 24 h)	Exert antioxidative effects; increase OCR, decrease ECAR, inhibit aerobic glycolysis; disrupt Warburg effect	↑SOD, ↑CAT, ↑GSH, ↓MDA, ↑acetyl-CoA, ↓LDH, ↓lactate, ↓glucose transporter 1 (GLUT-1), ↓LDHA, ↓p-Akt, ↓p-mTOR, ↓PKM2, ↓HIF-1α, ↓TNF-α, ↓IL-1β	
Inflammation related other diseases						
BV	Allergic chronic rhinosinusitis	Ovalbumin (i.p.) and *Staphylococcus aureus* enterotoxin B (intranasally)	Female BALB/c mice (0.5 and 5 ng/ml, intranasally, 3 times a week for 8 weeks)	Decrease neutrophils and eosinophils in nasal lavage fluid; decrease inflammatory cell infiltration and PAS-positive cells	↓INF-γ, ↓NF-κB, ↓AP-1	Seung-Heon Shin et al. (2018)
BV	Pleurisy	Carrageenan (injected into pleural cavity)	Male Balb/c mice (0.8 and 0.08 mg/kg, s.c. into the left Chize acupoint, LU-5, the first was 5 min before and a second was 12 h after carrageenan injection)	Suppress pleural exudate volume and leukocyte accumulation, increase the number of Fos-ir neurons	↓MPO, ↓IL-1β	Choi et al. (2018)
BV	Inflammatory periodontitis	*Porphyromonas gingivalis* (applied to gingival margin)	Male Balb/C mice (1, 10, and 100 μg/kg, once a week for 4 weeks)	Attenuate bone resorption and osteoclast formation	↓TNF-α, ↓IL-1β	Gu et al. (2019)
	Osteoclastogenesis	RANKL	Mouse monocyte/macrophage RAW 264.7 cells (1, 10, and 100 ng/ml, for 5 days)	Show no cytotoxic effect; inhibit osteoclastogenic differentiation; attenuate F-actin ring formation and osteoclast resorptive activity	↓Nuclear factor of activated T cells 1 (NFATc1), ↓integrin αv, ↓integrin β3, ↓cathepsin K, ↓TNF-α, ↓IL-1β, ↓p-Akt, ↓p-ERK 1/2, ↓p-p38, ↓p-JNK	
MEL	Periodontitis	PgLPS	HaCaT cells (0.1, 0.5, and 1 μg/ml, for 8 h)	Anti-inflammatory effects	↓IFN-γ, ↓TNF-α, ↓TLR-4, ↓IL-8, ↓IL-6, ↓pIκB, ↓NF-κB p65, ↓pAkt, ↓pERK1/2	Woon-Hae Kim et al. (2018)
BV	Allergic inflammatory disorders	PMA and A23187 (PMACI)	HMC-1 cells (5 and 10 μg/ml, for 30 min-24 h)	Suppress histamine release	↓TNF-α, ↓IL-6, ↓IL-1β, ↓p-ERK, ↓p-JNK, ↓p-p38, ↓p-MEK1/2, ↓p-MAPK kinase 3/6 (p-MKK3/6), ↓p-MKK4, ↓p-STAT3 (Ser727), ↓p-Akt	Kang et al. (2018)
		Compound 48/80 (i.p., Anaphylactic shock)	ICR male mice (20 mg/kg, i.p.)	Reduce mortality rates	↓TNF-α, ↓IL-6, ↓IL-1β, ↓p-ERK, ↓p-JNK, ↓p-p38, ↓p-STAT3 (Tyr705)	

Abbreviations are as shown in the literature. (*↓*), down-regulation or inhibition; (*↑*), up-regulation or activation.

### Effects on atopic dermatitis

As a chronic skin inflammatory disease, atopic dermatitis is caused by several genetic, inflammatory, and immunological abnormalities and characterized by recurrent eczema and itching (Gu et al., 2018; Lee Y. J. et al., 2020). BV and its main components, MEL and bvPLA_2_, have anti-inflammatory and atopic dermatitis effects *in vivo* and *in vitro*.

*In vivo*, BV treatment significantly reduced phthalic anhydride-induced atopic dermatitis clinical score, back and ear epidermal thickness, and the lymph node weight, decreased the level of IgE, IL-4, IL-13, TNF-α, IL-1β, and IL-6 and the number of eosinophil, neutrophil, monocytes, mast cells, F4/80-positive cells and Ly6G-positive cells, as well as inhibited the iNOS and COX-2 expression and activated MAPK and NF-κB in the skin tissues. *In vitro*, BV also suppressed MAPK and NF-κB pathway in RAW 264.7 murine macrophages treated with LPS and HaCaT human keratinocytes treated with TNF-α-/IFN-γ (Lee Y. J. et al., 2020). Besides MAPK and NF-κB pathways, Janus kinase (JAK)/STAT signal pathways were also blockaded by the treatment with BV or MEL in TNF-α/IFN-γ-treated HaCaT cells, and the chemokines CCL22 and CCL17 and pro-inflammatory cytokines IL-6, IL-1β, and IFN-γ decreased (An et al., 2018).

In addition, BV relieved atopic dermatitis by inactivating the complement system. *In vitro*, BV inhibited the complement system via the induction of CD55 by activating ERKs in THP-1 cells. *In vivo*, BV attenuated skin condition, and decreased clinical skin score in mice with atopic dermatitis-like lesions from 2.5% 2,4-dinitrochlorobenzene (DNCB) treatment; furthermore, BV and MEL inactivated the complement system, especially via the CD55 induction, which downregulated the membrane attack complex (MAC) and C3 convertase (C3C) levels (Kim Y. et al., 2019).

The bvPLA_2_ treatment suppressed atopic dermatitis-related skin swelling, improved ear thickness, reduced IgE and the expression of Th1 and Th2 cytokines (IFN-γ, IL-4, IL-6 and IL-10), induced Treg, and decreased epidermal and dermal thickness and macrophage and mast cell infiltration in *Dermatophagoides farinae* extract/DNCB-treated mice. However, the effects were eliminated in CD206^−/−^ mice after *D. farinae* extract/DNCB treatment. The above data suggested that bvPLA_2_ could alleviate atopic skin inflammation through interaction with CD206 (Shin D. et al., 2018).

### Effects on arthritis

Arthritis has a high incidence rate in the world and includes more than 100 forms, and the most common ones include RA, osteoarthritis, psoriatic arthritis and inflammatory arthritis (Tang, 2019). BV and its main components, MEL and apamin, and wasp venom, have anti-inflammatory, antioxidative, anti-apoptotic, and other effects on arthritis.

RA is a chronic inflammatory autoimmune disease, which is often affected by genetic, epigenetic, and environmental factors (Giannini et al., 2020). BV treatment decreased the paw volume and arthritis index in RA rats induced by Freund’s complete adjuvant, and showed anti-oxidant, anti-inflammatory, anti-genotoxic, and immunomodulatory effects through decreasing total oxidant status, oxidative stress index, myeloperoxidase (MPO), IL-1β, IL-6, TNF-α, TGF-β1 and mononuclear leukocyte DNA damage levels, and increasing total antioxidant status level (Kocyigit et al., 2019). In addition, wasp venom dramatically inhibited the viability of MH7A human RA synovial fibroblasts *in vitro* and induced MH7A cell apoptosis through up-regulating the expression of Caspase-3 and Bax and down-regulating that of Bcl-2 (Liu et al., 2021). Besides, bvPLA_2_ showed significant anti-inflammatory and arthritic effect on a RA mouse model though the induction of Tregs (Choi et al., 2021). Polymeric microneedle-mediated transdermal delivery of MEL inhibited the progression of RA in arthritis rat and mouse models, suppressed the levels of IL-17 and TNF-α, and raised the percentage of regulatory CD4 T cells, which suggested that polymeric microneedle-mediated MEL transdermal delivery could be used as a new treatment for RA and other autoimmune diseases (Du et al., 2021).

In addition, MEL showed anti-inflammatory effects on C518 rat knee joint cells induced by IL-1β *via* suppressing the expression of iNOS and preventing the degradation of IκB in cytoplasm and the migration of NF-κB from cytoplasm to nucleus, which suggested that MEL could be used as an anti-inflammatory candidate against osteoarthritis (Tang et al., 2021). BV and apamin treatment decreased the inflammatory paw edema, and reversed the alternation in weight-bearing distribution in monosodium urate (MSU)-administered mice by inhibiting proinflammatory cytokine production and inflammasome formation, as well as decreased MSU crystal formation, which suggested that BV and apamin could be potential novel agents for gouty arthritis (Lee Y. M. et al., 2020). Besides, BV also showed anti-inflammatory effect to alleviate pain and edema in gouty arthritis rats through reducing the expression of proinflammatory cytokines and chemokines (Goo et al., 2021).

### Effects on inflammation related digestive diseases

BV and MEL exerted anti-inflammatory effects on inflammation related digestive diseases, such as gastric ulceration, nonalcoholic steatohepatitis, ulcerative colitis, acute liver failure, etc.

BV showed gastro-protective effects against rats gastric ulceration induced by acetylsalicylic acid. It attenuated the haematological, haemostatic, and histopathological alterations, reduced the tissue eosinophil level, as well as decreased the ulcer index, fluid volumes, and pepsin concentrations in acetylsalicylic acid-treated rats, which attributed to its anti-inflammatory and antioxidant effects by decreasing TNF-α, MPO levels and MDA concentration, and increasing SOD activity and glutathione (GSH) concentration, its anti-apoptotic property by downregulating Bax and Caspase-3 levels, as well as protection of gastric tissue by upregulating Hsp70 (Mohamed et al., 2019). Besides, BV possessed antioxidant, anti-inflammatory, and anti-hyperlipidemic effects against rats nonalcoholic steatohepatitis induced by fructose, which may be due to the enhanced expression of nuclear receptors, liver X receptor α (LXRα) and farnesoid X receptor (FXR), the reduced liver sterol regulatory element-binding protein 1/2 (SREBP1/2) levels, and the improved intestine tight junction protein expression (Abd El-Haleim, 2020).

MEL showed antiulcerogenic effect in acetic acid-induced ulcerative colitis in mice. It increased the body weight gain, decreased colon mass index and preserved colon mucosa and submucosa, which could be via mitigating TLR4/TNF receptor associated factor 6 (TRAF6) mediated NF-κB and p38 MAPK signaling (Ahmedy et al., 2020). Besides, MEL improved survival rate, reduced serious symptoms and signs, and relieved hepatic inflammation by reducing total bilirubin, alanine transaminase (ALT), aspartate aminotransferase (AST), TNF-α and IL-1β levels in acute liver failure mice. In LPS-stimulated RAW264.7 macrophages, MEL exerted antioxidant and anti-inflammatory effects. Furthermore, MEL disrupted the Warburg effect by inhibiting the Akt/mTOR/pyruvate kinase M2 (PKM2)/HIF-1α pathway to alleviate inflammation *in vitro* and *in vivo*. Molecular docking analysis confirmed that MEL targeted PKM2, and knockout of PKM2 produced anti-inflammatory effects similar to MEL *in vitro*. The above findings highlighted a new strategy of MEL in treating acute liver failure (Fan et al., 2020).

### Effects on inflammation related other diseases

BV and its main components, including MEL, bvPLA_2_ and apamin, also exerted anti-inflammatory effects on other diseases, such as allergic chronic rhinosinusitis (Shin S. H. et al., 2018), pleurisy (Choi et al., 2018), inflammatory periodontitis (Kim W. H. et al., 2018; Gu et al., 2019), allergic inflammatory disorders (Kang et al., 2018), etc. For instance, BV showed anti-inflammatory effects in an allergic chronic rhinosinusitis mouse model. BV decreased neutrophil and eosinophil counts in nasal lavage fluid, reduced interferon-γ (INF-γ) level, inflammatory cell infiltration and PAS-positive cells, as well as suppressed NF-κB and activator protein-1 (AP-1) expressions in mouse nasal mucosa, which indicated that BV may possess potential value in treating allergic chronic rhinosinusitis (Shin S. H. et al., 2018).

## Analgesic effect

BV is usually used to relieve pain, mainly by injecting it into acupoints, such as ST36 (Zusanli) (Li et al., 2020a). In recently 4 years, BV and its main components, including MEL and bvPLA_2_, have been reported to attenuate neuropathic pain, osteoarthritis pain, and burn injury pain, etc., as shown in Table 4.

**TABLE 4:** Summary of the analgesic effects and mechanisms of BV and its main components.

BV and/or its components	Model	Inducer/Method	Animal/Cell (BV etc. administration)	Effects	Mechanisms	Reference
Neuropathic pain						
BV	Neuropathic pain	Oxaliplatin (i.p.)	SD rats (0.1 and 1 mg/kg, s.c., acupoint ST36 or LI11)	Alleviate cold and mechanical allodynia	Increase action potential and current threshold in acutely dissociated dorsal root ganglia neurons	Ji Hwan Lee et al. (2020)
MEL	Peripheral neuropathic pain	Oxaliplatin (i.p.)	SD rats (0.5 mg/kg, s.c., ipsilateral acupoint, Zusanli (ST 36) of right leg)	Alleviate mechanical and cold allodynia, inhibit hyperexcitated spinal WDR neuronal activity	Spinal α_1_- and α_2_- adrenergic receptors are involved in analgesic effects	Choi et al. (2019)
BV	Peripheral neuropathic pain	Vincristine (i.p.)	Male SD rats (1.0 mg/kg, s.c., right hind limb ST36 (Zusanli) acupoint)	Ameliorate cold and mechanical hypersensitivity, ameliorate hyperexcitation of spinal WDR neuron	Analgesia was mediated by descending noradrenergic pathway	Li et al. (2020a)
BV and venlafaxine (VLX)	Peripheral neuropathic pain	Paclitaxel (i.p.)	Male C57BL/6j mice (0.25, 1.0, and 2.5 mg/kg, s.c., ST36 (Zusanli acupoint) of right hind limb (BV); 1.0 mg/kg, s.c. (BV) and 40 mg/kg, i.p. (VLX))	Ameliorate cold and mechanical allodynia (co-administration > monotherapy)	Spinal α_2_-adrenergic receptor, 5-HT_1_, 5-HT_2_, and 5-HT_3_ receptors mediate analgesic effects (co-administration)	Li et al. (2020b)
bvPLA_2_	Neuropathic Pain	Right L5 SNL surgery	SD rats (0.2 mg/kg/day, i.p., for 2 days)	Attenuate mechanical allodynia	Analgesic effects are mediated by activation of spinal α_1_-adrenergic receptors	Woo et al. (2019)
Other pain						
BV	Osteoarthritis	Collagenase (intra-articularly injected)	Male Sprague-Dawley rats (1 and 2 mg/kg, ST-36 acupoint)	Improve pain-related behavior	Partial involvement of the δ-opioid and α_2_-adrenergic receptors	Huh et al. (2018)
BV	Burn injury	Right hind paw immersed in hot water (65°C, 3 s)	Male ICR mice (0.01, 0.02, and 0.1 mg/kg, s.c., ipsilateral knee area, once daily for 14 days)	Reduce mechanical allodynia, recover ipsilateral paw print area and single stance, reduce tissue damage	↓Sub P (dorsal root ganglia and spinal cord)	Kang et al. (2021)

Abbreviations are as shown in the literature. (*↓*), down-regulation or inhibition; (*↑*), up-regulation or activation.

### Effects on neuropathic pain

Neuropathic pain is a chronic pain, often caused by damaged or abnormally discharged neurons in the peripheral or central nervous systems (Hamad et al., 2018). Some anti-cancer agents, such as oxaliplatin, vincristine and paclitaxel, could induce neuropathic pain. BV acupuncture alleviated cold and mechanical allodynia induced by oxaliplatin through regulating the action potential threshold of A-fiber dorsal root ganglia neurons (Lee J. H. et al., 2020). Besides, s.c. injection of MEL at acupoint ST36 relieved the mechanical and cold allodynia, and inhibited hyperexcitated spinal wide dynamic range (WDR) neuronal activity, via activating the spinal α_1_-and α_2_-adrenergic receptors (Choi et al., 2019). BV acupuncture inhibited abnormal hyperexcitation of the spinal WDR neurons caused by cutaneous cold and mechanical stimulation in rats treated with vincristine, which was mediated by the descending noradrenergic pathway (Li et al., 2020a). Co-treatment of BV and venlafaxine produced a lasting and additive analgesic effects on paclitaxel-induced cold and mechanical allodynia in mice, through spinal α_2_-adrenergic, 5-HT_1_/5-HT_2_, and 5-HT_3_ receptors, providing a promising clinical strategy (Li et al., 2020b).

### Effects on other pain

BV acupuncture improved the pain-related behavior in the rat model of collagenase-induced osteoarthritis resulting from the partial partaking of the δ-opioid and α_2_-adrenergic receptors (Huh et al., 2018). Besides, BV biotherapy could sustainedly improve knee osteoarthritis pain and body function in a phase III clinical trial (Conrad et al., 2019).

Repeated BV treatment reduced the mechanical allodynia, recovered the ipsilateral paw print area and single stance, and reduced the tissue damage in a mice pain model induced by scalding burn. And BV stimulation inhibited the increase of Sub P expression in peripheral and central nervous systems. The above results suggested that BV treatment could effectively control the pain of burn patients (Kang et al., 2021).

## Anti-infectivity effect

In recent 4 years, BV and main components, MEL, bvPLA_2_, melectin and MPX, have been reported to show anti-infectivity effects, as shown in Table 5. In particular, the effects on severe acute respiratory syndrome–coronavirus 2 (SARS-CoV-2) and drug resistant bacteria have attracted more and more attention.

**TABLE 5:** Summary of the anti-infectivity effects and mechanisms of BV and its main components.

BV and/or its components	Model	Inducer/Method	Animal/Cell (BV etc. administration)	Effects	Mechanisms	Reference
Antiviral effect						
MEL, Sitagliptin- MEL optimized nanoformula	SARS-CoV-2	SARS-CoV-2	Vero E6 cells (1 ng/ml-10 μg/ml)	Show antiviral activity and *in vitro* Mpro 3CL-protease inhibition	—	Al-Rabia et al. (2021)
BV	HCV	HCV (JFH1 strain of genotype 2a)	Huh7it-1 cells (0.01–1000 ng/ml)	Inhibit HCV	Act on HCV particles directly	Sarhan et al. (2020)
CH/AL-BV	PRRSV	PRRSV type II LMY strain (intramuscularly)	Pigs (2 mg, nasally)	Reduce lung lesion scores and microscopic lesion score, decrease viral genome levels, increase virus neutralization titers levels	↑Th cells, ↑IL-12, ↑IFN-γ, ↑PRRSV-specific IgG Ab, ↑CD4^+^ T cells, ↑STAT4, ↑T-bet, ↓Treg cells, ↓IL-10, ↓TGF-β, ↓STAT5, ↓Foxp3, ↑IFN-γ secreting CD4^+^ T cells, ↑Th/memory cells	Lee et al. (2018)
Antibacterial effect						
BV	MRSA pneumonia	—	ATCC 33591 (reference strain, HA-MRSA) (0.98, 1.95, 3.9, and 7.8 μg/ml, for 4 h)	Show antibacterial activity	↓*hla*, ↓*agrA*, ↓α-hemolysin	Kong et al. (2020)
		—	USA300 (wild-type LAC strain, CA-MRSA)	Show antibacterial activity	—	
		MRSA	264.7 RAW macrophages (0.98, 1.95, 3.9, and 7.8 μg/ml)	—	↓TNF-α, ↓IL-6	
		USA300	A549 cells (0.78–6.25 μg/ml)	—	↓LDH	
		USA300 (intranasal infection)	Balb/c mice (0.125, 0.25, and 0.5 mg/kg/day, i.p.)	Increase survival rates; no abscesses, less lung edema and fewer inflammatory cells; reduce the number of bacteria in the lungs	↓Serum amyloid A3 (Saa3), ↓chemokine (C–X–C motif) ligand 9 (Cxcl9), ↓orosomucoid 1 (Orm1), ↑PON1	
BV and MEL	MRSA	—	Enterotoxigenic *S. aureus* isolates (ATCC 13565, ATCC 14558, ATCC 19095, and ATCC 23235) (0.45–917 μg/ml)	Show antibacterial activity, do not interfere with staphylococcal enterotoxin production or release	—	Pereira et al. (2020)
			MRSA ATCC 33591 and 5 clinical isolates (0.45–917 μg/ml)	Show antibacterial activity, exhibit bactericidal synergism with oxacillin. Show cell distortion, cell disintegration with leakage of cytoplasmic content and loss of cytoplasm content (ATCC 33591)	—	
BV, MEL and PLA_2_	*Escherichia coli*	—	*Escherichia coli* (ATCC^®^ 25922™) (0–200 μg/ml (BV), 0–10 μg/ml (PLA_2_))	Inhibit membrane-bound *E. coli.* F_1_F_0_-ATPase	—	Nehme et al. (2020)
BV	*Salmonella* strains	—	16 *Salmonella* strains (32–4096 μg/ml, for 24 h)	Show antibacterial activity, reduce biofilm formation	↑*rprA*, ↑*oxyS*, ↑*dsrA* (*S*. Newport Lhica N2 and *S*. Typhimurium T22)	Arteaga et al. (2019)
BV	Pathogenic Gram-positive and Gram-negative bacteria	—	*S. aureus* subsp. *aureus* (DSM 20231), *B. cereus* (DSM 6791), *S. enterica* subsp. *enterica* (DSM 14221), *L. innocua* (ATCC 3309) and *E. coli* (ATCC 25922) (12.5–800 μg/ml, for 24 or 48 h)	Show a broad spectrum of anti-infectivity properties	—	Tanuwidjaja et al. (2021)
Crude BV in Thailand	Pathogenic Gram-positive and Gram-negative bacteria, a fungus	—	*B. subtilis*, *M. luteus*, *S. aureus*, *S. aureus* MRSA, *S. epidermidis*, *E. coli*, *K. pneuminiae*, *S. typhimurium* (3.12–400 μg/ml, for 24 h); *C. albicans* (3.12–400 μg/ml, for 48 h)	Show more robust antibacterial activities against Gram-positive than Gram-negative bacteria or a fungus	—	Maitip et al. (2021)
MEL	XDR *Acinetobacter* spp.	—	XDR *Acinetobacter* spp. (0.0625–8 μg/ml)	Show antibacterial activity, cause fluorescence release from XDR isolates, induce aggregation, cell lysis, membrane disruption, and vesicle	—	Pashaei et al. (2019)
		*A. baumannii* ATCC 19,606 (inoculated on burn wound area)	Mouse model of third degree burn (Balb/c mice) (8, 16, and 32 μg/ml, incubated on infected area, for 2 h)	Eradicate colonized XDR bacteria	—	
MEL and doripenem	MDR *A. baumannii*	—	MDR *A. baumannii* (for 24 h)	Show synergistic antibacterial activity	—	Akbari et al. (2019)
MEL-doripenem and MEL-ceftazidime	MDR *P. aeruginosa*	—	MDR *P. aeruginosa* (for 24 h)	Show synergistic antibacterial activity	—	
Melectin	Broad-spectrum	—	*S. aureus* ATCC 25923, *P. aeruginosa* KCTC 2004, *K. pneumoniae* KCTC 2008, *E. coli* ATCC 25922; drug resistant strains (0–32 μM)	Show antibacterial activity	Have an α-helical structure, bind to LPS or LTA and kill bacteria through bacterial membrane permeabilization (*S. aureus* ATCC 25923, and *P. aeruginosa* ATCC 27853)	Ko et al. (2020)
		*S. aureus*	Human dermal fibroblasts (5 μM, for 24 h)	—	↓TNF-α, ↓IL-8, ↓IL-6, ↓IL-1β	
Melectin	Broad-spectrum	—	Drug-sensitive bacterial strains (4, 8, and 16 μM, etc.)	Show antibacterial activity	Have an α-helix form, effect on the outer and inner membrane of bacteria (*E. coli*), interact with DNA	Liang et al. (2021)
MPX	*Actinobacillus pleuropneumoniae*	—	*A. pleuropneumoniae* (16, 32, and 64 μg/ml)	Show antibacterial activity, increase membrane permeability, release bacterial proteins and Ca^2+^, Na^+^ and other cations, reduce biofilm formation	↑PurC, ↓ApxI, ↓ApxII, ↓Apa1, ↑Sap A	Wang et al. (2020)
		*A. pleuropneumoniae* serotype 5b L20 strain (inoculated intranasally)	Female BALB/c mice (20 mg/kg, i.p.)	Protect mice from a lethal dose of *A. pleuropneumoniae*, decrease the number of bacteria colonising the lung, relieve lung inflammation	↓TNF-α, ↓IL-6	
Antifungal effect						
BV	*Trichophyton rubrum*	—	*T. rubrum* (0.1, 10, and 40 mg/distilled water 100 μL)	Deliver a significant level of inhibition	—	Park et al. (2018)

Abbreviations are as shown in the literature. (*↓*), down-regulation or inhibition; (*↑*), up-regulation or activation.

### Antiviral effect

Coronavirus disease (COVID-19), caused by SARS-CoV-2, has infected people in 210 countries and was declared a pandemic by WHO on 12 March 2020 (Al Naggar et al., 2021). BV has shown potent antiviral, anti-inflammatory and immunomodulatory effects, which suggested that BV treatment could be a promising complementary therapy to prevent SARS-CoV-2 (Kasozi et al., 2020; Al Naggar et al., 2021; Lima et al., 2021). In Hubei Province in China, the local beekeeper association investigated 5115 beekeepers (including 723 in Wuhan) from 23 February to 8 March in 2020. The results showed that no one had any symptoms related to COVID-19 (Yang et al., 2020). In addition, sitagliptin-MEL optimized nanoformula was repurposed, and showed antiviral potential against SARS-CoV-2 isolate with IC_50_ values of 8.439 µM and *in vitro* 3CL-protease inhibition with IC_50_ values of 7.216 µM. Sitagliptin-MEL could ensure improved delivery to target cells and cell uptaking, with anti-SARS-CoV-2 activity (Al-Rabia et al., 2021).

Human immunodeficiency virus (HIV) has been a major social and health problem for about 30 years. BV was safe and maybe therapeutic in a specified dose, owing to MEL uptake by HIV infected cells which resulted in reduced HIV gene expression and replication (Uzair et al., 2018). A recent study using bioinformatics tools indicated that in addition to envelope and long terminal repeat, HIV capsid and proteases could have great potential as MEL targets (Dehghani et al., 2020).

Hepatitis C virus (HCV) infection is a worldwide health concern, which can lead to serious liver diseases. BV inhibited the entry stages of HCV infection cycle by interacting with virus particles, through a non-common peptide(s), which demonstrated that the BV could be a potential candidate for characterizing and developing new natural anti-HCV agents (Sarhan et al., 2020).

Besides, chitosan/alginate nanoparticle encapsulated BV (CH/AL-BV) was developed, and nasal administration of CH/AL-BV induced non-specific immune stimulating effects, and protected against porcine reproductive and respiratory syndrome virus (PRRSV) infection, suggesting that nasal-delivered CH/AL-BV could produce effective protective immunity against PPRSV infection (Lee et al., 2018).

### Antibacterial effect

BV and MEL showed antibacterial activity against MRSA (Kong et al., 2020; Pereira et al., 2020). *In vitro*, BV showed strong anti-infectivity effects against two MRSA strains, ATCC 33591 and USA300, and decreased the *hla* and *agrA* genes expression and the α-hemolysin production in ATCC 33591. BV alleviated the secretion of TNF-α and IL-6 in MRSA-stimulated 264.7 RAW macrophage. Besides, BV protected A549 cells from MRSA. *In vivo*, BV increased mouse survival rates, decreased the bacteria number in the lungs and relived symptoms of pneumonia induced by MRSA in mice. The above results indicated that BV could be a candidate for treating pneumonia caused by MRSA infection (Kong et al., 2020). BV and mainly MEL and bvPLA_2_, could inhibit *E. coli* F_1_F_0_-ATPase enzyme, a key molecular target for bacteria survival, which could be regarded as important candidate drugs against resistant bacteria (Nehme et al., 2020).

Besides, BV showed anti-infectivity activity against 16 *Salmonella* strains, and the minimum inhibitory concentrations (MIC) ranged from 256 to 1024 μg/ml. In addition, BV inhibited biofilm formation in 14 strains. The above findings indicated that BV was a potential anti-infectivity agent against food-borne pathogens (Arteaga et al., 2019).

Bacterial infection from antibiotic resistant bacteria has become a main cause of morbidity and mortality after burns. *In vitro*, MEL showed antibacterial activity against extensively drug-resistant (XDR) *Acinetobacter* spp., caused fluorescence release from XDR isolates which severed as a marker of membrane damage, and had membranolytic effect. *In vivo*, MEL eradicated the colonized XDR bacteria on infected mouse model with third-degree burn, and no toxicity at the therapeutic dose was observed (Pashaei et al., 2019). Furthermore, MEL-doripenem showed synergistic antibacterial activity against MDR *A. baumannii* isolates, while MEL-doripenem and MEL-ceftazidime showed synergistic antibacterial activity against multidrug-resistant (MDR) *P. aeruginosa* (Akbari et al., 2019). The above results showed that MEL, MEL-doripenem and MEL-ceftazidime could be regarded as topical drugs against burn infections from antibiotic resistant bacteria.

Melectin, from the venom of a bee, *Melecta albifrons*, showed broad-spectrum anti-infectivity activities against standard sensitive/clinical drug-resistant bacteria strains, and exhibited low or moderate cytotoxicity and no hemolytic activity. Besides, melectin reduced the release of TNF-α, IL-1β, IL-6, and IL-8 from *S. aureus*-stimulated human fibroblasts. These findings suggested that melectin could be considered as a new template against multidrug resistance (Ko et al., 2020; Liang et al., 2021).

MPX, from wasp venom, showed good antibacterial effect on *Actinobacillus pleuropneumoniae*, a causative agent of respiratory infections in pigs. MPX destroyed the bacterial cell membrane, blocked the bacterial biofilms formation, and modulated the expression of virulence factors of *A. pleuropneumoniae in vitro*, as well as protected mice from a lethal dose of *A. pleuropneumoniae* and relieved lung inflammation *in vivo*, which provided information for the clinical application of MPX (Wang et al., 2020).

### Antifungal effect

The antifungal effects of BV components on *Trichophyton rubrum* have been evaluated, and the results showed that BV provided a significant level of inhibition in its overall form, and BV based products may play a potential antifungal therapeutic role (Park et al., 2018).

## Protect effect on liver, kidney, lung and muscle injury

### Protect effect on liver injury

BV and its main components, such as MEL and bvPLA_2_, showed protect effects on liver diseases, including non-alcoholic fatty liver, hepatoxicity and cholestatic liver disease, as shown in Table 6.

**TABLE 6:** Summary of the protect effects and mechanisms of BV and its main components on liver, kidney, lung and muscle injury.

BV and/or its components	Model	Inducer/Method	Animal/Cell (BV etc. administration)	Effects	Mechanisms	Reference
Liver injury						
BV	Non-alcoholic fatty liver	HFD	Male Wistar rats (0.01, 0.05, 0.1 mg/kg, s.c.)	Normalize lipid profile values, decrease insulin resistance index, increase GSH/glutathione disulphide (GSSG) ratio; improve the architecture of liver cells showing normal sinusoids	↓ALT, ↓AST, ↓gamma-glutamyltransferase, ↓bilirubin, ↓FBG, ↓insulin, ↓TG, ↓TC, ↓LDL-C, ↓hepatic TGs, ↓hepatic TC, ↑HDL-C, ↑hepatic GSH, ↓serum GSSG, ↑glutathione reductase, ↑GST, ↑tGPx, ↑sGPx, ↑nsGPx, ↑Nrf2, ↓MDA, ↑adiponectin, ↓TNF-α	Hanafi et al. (2018)
BV and honey	Hepatoxicity	LPS and CCl_4_ (i.p.)	Adult male albino rats (1 mg/kg bw, i.p. (BV); 1 mg/kg bw, i.p. and 25 mg/kg bw, orally (BV and honey); every day for 2 months)	Provide a protective effect on hepatotoxicity, reestablish disturbed hematological parameters and liver histopathology	↓ALT, ↓AST, ↓alpha-fetoprotein (AFP), ↓Plasma lipid peroxide, ↑GPx	Meligi et al. (2020)
MEL	Hepatotoxicity	Isoniazid and rifampicin (p.o.)	Male rats *Rattus rattus* (15 and 30 μg/kg, s.c., for 15 days)	Increase body weight and liver weight, decrease total protein content, show high hematological alterations, decrease severe histopathological lesions	↓Direct bilirubin, ↓total bilirubin, ↓alkaline phosphatase (ALP), ↓ALT, ↓AST, ↓LDH, ↑GSH	Naji et al. (2021)
bvPLA_2_	Cholestatic liver disease	3,5-diethoxycarbonyl-1,4-dihydrocollidine diet	Male C57BL/6N mice (0.2 mg/kg, i.p., twice a week for 4 weeks)	Ameliorate cholestatic liver injury and fibrosis, attenuate apoptotic cell death, increase the number of Foxp3-positive cells, decrease the number of F4/80^+^ macrophages and CD4^+^ T-cells	↓AST, ↓ALT, ↓ALP, ↓total bilirubin, ↓collagen I, ↓fibronectin, ↓α-SMA, ↓TGF-β1, ↓p-Smad2/3, ↓cleaved Caspase-3, ↓cleaved PARP-1, ↓TNF-α, ↓IL-6, ↓p-NF-κB p65, ↑IL-10	Jung-Yeon Kim et al. (2021b)
Kidney injury						
BV	Sepsis-related acute kidney injury	LPS (i.p.)	C57BL/6N mice (100 μg/kg bw, i.p.)	Reverse renal dysfunction and structural injury, reduce the number of Mac-2 or CD4-positive cells, decrease the number of TUNEL-stained cells	↓creatinine, ↓blood urea nitrogen (BUN), ↓neutrophil gelatinase-associated lipocalin (NGAL), ↓kidney injury molecule-1 (Kim-1), ↓TNF-α, ↓IL-6, ↓4-hydroxynonenal (4-HNE), ↓MDA, ↓cleaved Caspase-3, ↓cleaved PARP1, ↓p53	Jung-Yeon Kim et al., (2020a)
MEL	Sepsis-related acute kidney injury	LPS (i.p.)	Male C57BL/6 N mice (0.01 mg/kg, i.p.)	Dampen renal dysfunction and structural damage, suppress NF-κB DNA-binding activity, reduce Mac-2-positive and CD4-positive cells, reduce 4-HNE-positive cells, reduce the number of TUNEL-stained cells, enhance survival rate	↓creatinine, ↓BUN, ↓NGAL, ↓Kim-1, ↓TNF-α, ↓IL-6, ↓p-IκB-α, ↑IκB-α, ↓p-NF-κB p65, ↓NF-κB p65, ↓MDA, ↓NOX4, ↑Nrf2, ↑HO-1, ↑quinone oxidoreductase 1 (NQO1), ↓cleaved Caspase-3, ↓cleaved PARP-1, ↓p53, ↓Bax, ↓receptor-interacting serine/threonine protein kinase 1 (RIPK1), ↓RIPK3, ↓p- mixed-lineage kinase domain-like protein (MLKL)	Jung-Yeon Kim et al. (2021a)
Apamin	Sepsis-related acute kidney injury	LPS (i.p.)	Male C57BL/6N mice (0.1 mg/kg, i.p.)	Ameliorate renal dysfunction and histological injury, alleviate loss of brush borders in the proximal tubule, reduce 4-HNE-positive cells, increase GSH/GSSG ratio, decrease the number of TUNEL-stained cells, decrease number of galectin-3-stained and CD4-stained cells	↓creatinine, ↓BUN, ↓NGAL, ↓Kim-1, ↓MDA, ↓NOX4, ↑HO-1, ↓cleaved Caspase-3, ↓cleaved PARP-1, ↓TNF-α, ↓IL-6, ↓TLR4, ↓p-p65/p65, ↓E-selectin, ↓VCAM-1, ↓ICAM-1	Jung-Yeon Kim et al., (2020b)
MEL	Acute kidney injury	Cisplatin (i.p.)	Male C57/BL6 mice (10, 50, and 100 μg/kg, i.p., for 5 days)	Increase survival, ameliorate renal tubular damage, decrease CD16/32^+^ cells, increase Arg1^+^ cells, induce M2 macrophage infiltration into the kidney	↓creatinine, ↓BUN, ↓IL-6, ↓IL-1β, ↑IL-10, ↓COX-2, ↓macrophage receptor with collagenous structure (MARCO), ↑arginase 1	Hyunseong Kim et al. (2020)
Apamin	Renal fibrosis	Unilateral ureteral obstruction surgical procedure	Male C57BL/6 mice (0.5 mg/kg, i.p., twice a week)	Attenuate renal interstitial fibrosis, decrease F4/80^+^ macrophages	↑E-cadherin, ↓NGAL, ↓creatinine, ↓BUN, ↓TNF-α, ↓IL-1β, ↓cleaved-IL-1β, ↓IL-6, ↓MCP-1, ↓α-SMA, ↓vimentin, ↓fibronectin, ↓fibroblast-specific marker-1 (FSP-1), ↓TGF-β1, ↓p-STAT3, ↓p-Smad2, ↓p-Smad3, ↑Smad7	Gwon et al. (2021)
		TGF-β1	Rat renal interstitial fibroblast cells (NRK-49F) (0.5, 1, and 2 μg/ml, for 48 h)	Inhibit renal fibroblasts activation	↓collagen I, ↓fibronectin, ↓TGF-β1, ↓p-STAT3/t-STAT3, ↓p-Smad2, ↓p-Smad3, ↑Smad7	
Lung and muscle injury						
MEL	Lung injury	Paraquat (i.p.)	Male Swiss albino mice (0.1 and 0.5 mg/kg, i.p., twice per week for 4 weeks)	Reduce lung injuries	↑SOD, ↑CAT, ↑GPx, ↓MDA, ↓NO, ↑Bcl-2, ↑Survivin, ↓Ki-67	El-Aarag et al. (2019)
MEL	Muscle injury	Drop mass method	Male C57/BL6 mice (4, 20, and 100 μg/kg, i.m., for 7 days)	Increase locomotor activity in open field test and treadmill running activity, improve histological changes of damaged tissue	↓MCP-1, ↓TNF-α, ↓IL-6, ↑MyoD, ↑myogenin, ↑α-SMA, ↑MHC	Jae Eun Lee et al. (2019)
			C2C12 cells (1, 2.5, 5 ng/ml, for 16 h)	—	↑MHC	

Abbreviations are as shown in the literature. (*↓*), down-regulation or inhibition; (*↑*), up-regulation or activation.

The trend of elevated fasting glucose and insulin levels as well as lower levels of antioxidant parameters such as glutathione-S-transferase (GST), glutathione peroxidase (GPx), Nrf2 and GSH in rats induced by high-fat diet (HFD) was reversed by BV, and the levels of adiponectin and TNF-α were restored. The above results suggested that BV is a promising alternative treatment option for non-alcoholic fatty liver disease (Hanafi et al., 2018). BV and honey showed protective effects on hepatoxicity and lipid peroxidation in rats caused by LPS and carbon tetrachloride, while MEL possessed hepatoprotective activity on isoniazid- and rifampicin-induced liver hepatoxicity in rats, both of which suggested that a combination of BV and honey, or MEL could be beneficial for the prevention of hepatoxicity and could be used as potential therapeutic agents (Meligi et al., 2020; Naji et al., 2021). Besides, after treatment with bvPLA_2_, the levels of cholestasis markers in the serum were attenuated, the abnormal state of liver fibrosis was restored, and hepatocyte inflammation and apoptosis were inhibited in mice with diet feeding 3,5-diethoxycarbonyl-1,4-dihydrocollidine (Kim J.-Y. et al., 2021b).

### Protect effect on kidney injury

BV and its main components, such as MEL and apamin, showed protect effects on acute kidney injury and renal fibrosis, as listed in Table 6.

Acute kidney injury associated with sepsis is a worldwide health dilemma. Sepsis-related acute kidney injury is a global health issue. During sepsis, the endotoxin LPS mediates systemic inflammatory responses. BV, MEL and apamin ameliorated LPS-induced acute kidney injury *via* the suppression of oxidative stress, inflammation and cell death in mice (Kim J. Y. et al., 2020a; Kim J. Y. et al., 2020b; Kim et al., 2021a). Take MEL as an example, it dampened renal dysfunction and structural damage, and at the same time, the levels of direct tubular injury markers were attenuated in LPS-treated mice. MEL decreased systemic and renal TNF-α and IL-6 levels, attenuated the accumulation of immune cells in the kidney, and suppressed NF-κB pathway. MEL reduced MDA level and inhibited the expression of nicotinamide adenine dinucleotide phosphate oxidase 4 (NOX4), and promoted Nrf2-mediated antioxidant defenses, as well as inhibited apoptosis and necrosis after LPS treatment. In addition, the survival duration of LPS-injected mice was significantly prolonged after MEL injection. The above results suggest that MEL could have a responsibility among the prospective options for the prevention and treatment of renal complications of sepsis (Kim J.Y. et al., 2021b). Besides, MEL alleviated mice acute kidney injury induced by cisplatin via regulating M2 macrophage expression (Kim H. et al., 2020).

With the chronic accumulation of progressive renal fibrosis, the kidneys continuously suffer one-way, irreversible damage, and end-stage diseases such as renal failure arrive as scheduled. *In vivo*, apamin inhibited renal fibrosis induced by unilateral ureteral obstruction; *in vitro*, it resisted renal fibroblasts activation induced by TGF-β1. This peptide suppressed TGF-β1/Smad2/3 and STAT3 pathways to inhibit inflammation response and tubular atrophy as well as reduce the activation of myofibroblast and the expression of fibrotic gene. These studies suggest that apamin may have a competitive advantage in the screening of therapeutic agent alternatives against renal fibrosis (Gwon et al., 2021).

### Protect effect on lung and muscle injury

In addition, MEL reduced lung injuries induced by paraquat in mice. It increased SOD, CAT, and GPx activity, and decreased MDA and NO levels, as well as upregulated Bcl-2 and survivin expressions and downregulated Ki-67 expression, which demonstrated that MEL restored the pathological alternations of paraquat-induced lung injury in mice from the perspectives of enhancing antioxidant activity and reducing apoptosis (Table 6) (El-Aarag et al., 2019).

MEL ameliorated muscle function and histological changes of damaged tissue, and debilitated the production of pro-inflammatory factors, including IL-6, MCP-1, and TNF-α in muscle contusion mouse model. In addition, the expression of muscle recovery and regeneration-related biomarkers, such as myogenin, MyoD, α-smooth muscle actin (α-SMA) and myosin heavy chain (MHC), was elevated in mice. The results suggested that MEL could be a promising candidate for the muscle injury treatment (Table 6) (Lee J. E. et al., 2019).

## Protect effect on other diseases

### Anti-diabetic efficacy

Bee wax coated water-soluble fraction of BV (BWCBVA) and MEL-loaded NPs showed anti-diabetic efficacy in diabetic animal models, as shown in Table 7. BWCBVA decreased blood glucose level, normalized the serum biochemical parameters, and increased the body weight in streptozotocin-induced diabetic rats. Also, administration of BWCBVA reduced the expression of PI3K-p85 and enhanced the expression of glucokinase in liver, as well as increased the number of islet cells and decreased the damage of β-cell in pancreas. Furthermore, co-administering BWCBVA with nifedipine and nicorandil resulted in insulin secretion through enhancing the calcium ion influx and obstructing the potassium ion channel. The above results indicated that BWCBVA could be considered as an available mode to treat diabetes (Balamurugan et al., 2019). In addition, prolonged MEL release from polyelectrolyte-based nanocomplexes improved blood glycemic control in a type II diabetes mouse model (Gui et al., 2020).

**TABLE 7:** Summary of the protect effects and mechanisms of BV and its main components on other diseases.

BV and/or its components	Model	Inducer/Method	Animal/Cell (BV etc. administration)	Effects	Mechanisms	Reference
Diabetes						
BWCBVA	Diabetes mellitus	Streptozotocin (i.p.)	Male Wister albino rats (0.125, 0.25 and 0.50 mg/kg, orally, once a day for 3 weeks)	Decrease glucose level, increase body weight, increase the islet cell numbers and decrease β-cell damage, result in insulin secretion by enhancing the calcium ion influx and obstructing the potassium ion channel when co-administered with nifedipine and nicorandil	↑insulin, ↓HbA1c, ↓TC, ↓TG, ↓SGOT, ↓SGPT, ↓PI3K-p85, ↑glucokinase	Balamurugan et al. (2019)
MEL-loaded nanoparticles	Type II diabetes	—	Male db/db mice (3 mg/kg, i.p.)	Decrease blood glucose level and body weight	—	Gui et al. (2020)
Cardiovascular system						
BV	Cardiac dysfunction	Streptozotocin (i.p.), a high-fat diet	Male albino rats (0.5 and 1.23 mg/kg, i.p., daily for 4 weeks)	Increase bodyweight, decrease fasting blood glucose levels	↑insulin, ↓TG, ↓TC, ↓LDL-C, ↓VLDL-C, ↑HDL-C, ↓CK-MB, ↓LDH, ↓troponin I, ↑TAC, ↓MDA, ↓NF-κB, ↓VCAM-1, ↓galectin-3	Zahran et al. (2021)
bvPLA_2_	Atherosclerosis	Atherogenic high-cholesterol diet	ApoE^−/−^ mice (0.2 mg/kg, i.p., every 2 days after a 4-week diet)	Increase in CD4^+^ Foxp3^+^ Treg cells in the lymph nodes, reduce atherosclerotic lesions and foam cell formation	↑HDL-C, ↓LDL-C, ↑HDL/LDL, ↓AST, ↓IFN-γ, ↓TNF-α	Kang et al. (2020)
HFD-induced obesity						
BV	HFD-induced obesity	Adipocyte differentiation	3T3-L1 preadipocytes (2.5, 5, and 10 μg/ml, for 3 or 9 days)	Suppress cell hyperplasia and lipid accumulation	↓C/EBPα, ↓C/EBPβ, ↓C/EBPδ, ↓PPARγ, ↑p-ERK, ↑p-JNK, ↓p-p38, ↑p-AMPK, ↑p-ACC	Cheon et al. (2018)
		HFD	C57BL/6 mice (0.1 and 1 mg/kg, i.p., during the last 4 weeks)	Suppress body weight, fat, and lipid accumulation	↓PPARγ, ↓C/EBPα, ↑p-AMPK, ↑p-ACC	
bvPLA_2_	HFD-induced obesity	HFD	Male C57BL/6 mice (0.5 mg/kg, i.p., every 3 days for 11 weeks)	Reduce body weight and adipose tissue weight, decrease hepatotoxicity and nephrotoxicity, alleviate metabolic dysfunction; decrease macrophage infiltration and crown-like structures formation, decrease the M1/M2 ratio	↓ALT, ↓creatine, ↓glucose, ↓LDL-C, ↓insulin, ↓leptin, ↓TNF-α, ↓IL-12a, ↑IL-4, ↑CD206, ↓C/EBPα, ↑UCP-1	Jeong et al. (2021)
		LPS for M1 activation	Bone marrow cells (0–10 µg)	Inhibit M1 macrophage polarization	↓TNF-α, ↓IL-12a	
		IL-4 and IL-13 for M2 activation	Bone marrow cells (0–10 µg)	Stimulate M2 macrophage polarization	↑Ym1, ↑CD206	
		Adipocyte differentiation	3T3-L1 preadipocytes (0–10 µg)	Regulate lipid accumulation through macrophages	—	
BV	Obesity-associated inflammation	—	Differentiated 3 T3-L1 preadipocyte (0, 1, 5, and 10 μg/ml)	Inhibit lipid accumulation;	↓PPARγ, ↓C/EBPα	Hee-Yeon Kim et al. (2020)
		LPS	RAW264.7 macrophages (0, 1, 5, and 10 μg/ml)	—	↓TNF-α, ↓COX-2, ↓iNOS, ↓IL-1β	
		—	Differentiated 3 T3-L1 preadipocyte, RAW264.7 macrophages (0, 1, 5, and 10 μg/ml)	—	↓TNF-α, ↓COX-2, ↓iNOS, ↓IL-1β, ↓IL-6, ↓MCP-1, ↑AdipoQ, ↑GLUT4	
Wound healing						
BV	Impaired diabetic wound healing	Streptozotocin (i.p.), excising the skin and underlying panniculus carnosus	Male BALB/c mice (200 μg/kg/wounded area/day, s.c., for 15 days)	Enhance wound closure, rescue wound tissue macrophages from apoptosis and restore impaired functional activity,	↑Collagen I, ↑Ang-1, ↑Nrf2, ↑phospho-tyrosine (pTyr) Tie-2, ↑peNOS, ↑pAkt, ↑pERK, ↑CD31, ↑GSH Px, ↑mitochondrial SOD, ↑CAT, ↑CCL2, ↑CCL3, ↑CXCL2, ↑BD-2	Hozzeinab et al. (2018)
bvPLA_2_	Wound healing	Polyinosinic-polycytidylic acid [poly (I:C)]	HaCaT cells (0.05, 0.1, 0.5, 1 and 5 µg/ml)	Increase poly (I:C) localization in the lysosomes; increase the intracellular uptake of poly (I:C) and its enzymatic activity is essential	↑IL-8, ↑p-ERK, ↑p-JNK, ↑p-IKKαβ, ↑p-IκB-α	Nakashima et al. (2020)
Reproductive performance and growth performance						
BV	Reproductive performance	—	Male V-line rabbits (bucks) (0.1, 0.2, and 0.3 mg/rabbit, s.c., twice weekly over 20 weeks)	Show shorter reaction time (increased libido), increase viable sperm and concentration, total sperm output, live sperm, fertility percentage, testosterone concentration, and total protein, albumin, and glucose levels	↑TAC, ↑GST, ↑GSH, ↑IgA, ↑IgM	El-Hanoun et al. (2020)
BV	Reproductive performance	—	Spanish V-line mature does (primiparous) (0.1, 0.2 and 0.3 mg/rabbit, s.c., twice weekly for 1 week before mating and 4 weeks after mating)	Increase litter size at birth, litter weight, survival rate at weaning age, milk yield, conception and fertility rates, decrease plasma total lipids, cholesterol and urea	↑Estradiol 17-β (E2), ↓progesterone (P4), ↓AST, ↓ALT, ↑TAC, ↑GSH, ↑GST, ↑GPx, ↑SOD, ↓MDA, ↓TBARS, ↑IgG, ↑IgM, ↑IgA	Elkomy et al. (2021)
BV	Growth performance	—	Male broiler chicks (10, 50, 100, and 500 μg/kg of diet, for 5 weeks)	Improve feed conversion ratio, increase body weight gain; lower relative weight of spleen, bursa of Fabricius, and liver, increase relative breast meat yields; increase lightness (L*) value for meat decrease ileal villus height and narrowed width; reduce total in caecal digesta	↑Secretory IgA, ↓NO	Kim et al. (2018a)
Other aspects						
BV	Asthma	IL-13	A549 cells (0.1 and 1.0 μg/ml)	—	↓p-Akt/Akt, ↓SPDEF, ↑FOXA2, ↓MUC5AC	Sanga Kim et al. (2021)
BV	iPSCs	—	iPSCs (0–5 μg/ml)	Induce cell death, reduce cell membrane integrity; induce both apoptosis and necroptosis; enhance calcium influx, calpain activity, and ROS generation	↓F-Actin, ↓FAK, ↓Talin-1, ↓vinculin, ↑p-RIP, ↑p-MLKL, ↑cleaved Caspase-3, ↑cleaved PARP, ↑p-CaMKII, ↑p-cPLA_2_	Aeyung Kim et al. (2020)
BV alongside CS	Radial bone defect	A 5 mm bone piece from the middle of the radius bone was removed to produce a non-union model	Male Wistar rats (0.1 mg, injected percutaneously into the defect site)	Show high radiographic outcomes, density of osseous tissue, and osteocytes and osteoblasts count	—	Meimandi-Parizi et al. (2018)
Natural extract eye drops containing BV, musk, and deer antlers	Dry eye disease	Scopolamine (s.c.)	Male Sprague-Dawley rats (4 times a day for 5 days)	Decrease corneal fluorescein staining scores	↓LDH, ↑MUC5AC	Choi et al. (2020)
		Lacrimal gland-excised	Male Sprague-Dawley rats (4 times a day for 5 days)	Decrease corneal fluorescein staining scores, increase tear volume	↓LDH, ↑MUC5AC	
bvPLA_2_	Abortion	LPS (i.p.)	Mated female C57BL/6 mice (0.5 mg/kg, i.p., once a week for 2 weeks prior to mating)	Prevent fetal loss accompanied by growth restriction in the remaining viable fetus, increase CD4^+^ T helper cells and uNK cells, decrease CD8^+^ cytotoxic T cell populations	↓TNF-α, ↓IFN-γ, ↓IL-17A	Baek et al. (2019)

Abbreviations are as shown in the literature. (*↓*), down-regulation or inhibition; (*↑*), up-regulation or activation.

### Protective effect on cardiovascular system

Administration of BV decreased fasting blood glucose, serum total cholesterol (TC), triglycerides (TG), LDL-C, VLDL-C, creatine kinase (CK)-MB, LDH, troponin I, and heart MDA, NF-κB levels, and heart vascular cell adhesion molecule-1 (VCAM-1) and galectin-3 expression, as well as increased bodyweight, plasma insulin, serum HDL-C and heart total antioxidant capacity (TAC) levels in diabetic hyperlipidemic rats, which indicated that BV could reduce oxidative stress and downregulate NF-κB pathway to improve cardiac dysfunction (Zahran et al., 2021). Besides, bvPLA_2_ could be a potential agent for atherosclerosis through inducing Treg cells (Kang et al., 2020). The above results are shown in Table 7.

### Attenuating HFD-induced obesity

*In vitro*, BV suppressed lipid accumulation, and inhibited adipogenesis by reducing the expression of CCAAT/enhancer-binding proteins (C/EBPs), peroxisome proliferator-activated receptor γ (PPARγ), and p-p38, as well as by upregulating the expression of p-ERK, p-JNK, p-adenosine monophosphate–activated protein kinase (AMPK), and p-acetyl-coenzyme A carboxylase (ACC) during differentiation of 3T3-L1 preadipocytes. *In vivo*, BV inhibited fat and lipid accumulation and reduced body weight, through downregulating the expression of adipogenic markers, PPARγ and C/EBPα, and upregulating the expression of p-AMPK and p-ACC in HFD-induced obese mice, suggesting BV an effective preventive and therapeutic candidate for obesity (Cheon et al., 2018). Besides, bvPLA_2_ also attenuated HFD-induced obesity in mice (Jeong et al., 2021). In addition, BV could attenuate adipocyte hypertrophy, as well as improve insulin resistance and obesity-related inflammation in obese adipose tissue (Kim H. Y. et al., 2020). The above results are shown in Table 7.

### Improving wound healing

Diabetic wound formation is one of the serious complications of diabetes. BV treatment improved wound closure in streptozotocin-induced mice diabetic model through enhancing β-defensin-2 (BD-2) and collagen expression and restoring angiopoietin-1 (Ang-1) and Nrf2 levels, and thereby increasing the Tie-2 downstream signaling, including the phosphorylation of endothelial nitric oxide synthase (eNOS), Akt and ERK. Furthermore, BV restored wounded tissue antioxidant enzymes GSH Px, mitochondrial SOD and CAT activities, and the chemokines CCL2, CCL3 and CXCL2 levels, and then rescued wound macrophages from apoptosis induced by mitochondrial membrane potential. The above results provided a new example for BV treatment to stimulate angiogenesis and improve the wound healing process of diabetes (Hozzeinab et al., 2018). Besides, bvPLA_2_ increased IL-8 production in HaCaT cells induced by poly (I:C), upregulated the expression of p-ERK, p-JNK, p-IKKαβ and p-IκB-α. It enhanced the intracellular poly (I:C) uptake, and the enzymatic activity was essential. These findings indicated that bvPLA_2_ could ameliorate wound healing via the enhancement of TLR3 responses (Nakashima et al., 2020). The above results are shown in Table 7.

### Improving reproductive performance and growth performance

BV could improve reproductive performance and growth performance, as shown in Table 7. BV improved reproduction and immune response of male and female rabbits, which demonstrated that BV could be used as an effective and safe substitute for artificial chemical drugs (sex stimulants) in rabbit breeding to improve some reproductive characteristics, immune response and health (El-Hanoun et al., 2020; Elkomy et al., 2021). Besides, dietary BV improved growth performance, the yield and quality of breast meat, and the concentration of secretory IgA on ileal mucosa in broiler chickens, and lowered internal organs including spleen, bursa, and liver, caecal short-chain fatty acids concentrations and ileal villus height, which demonstrated that BV could be regarded as a natural value-added substitute for antibiotics in feed to enhance growth and animal health (Kim D. H. et al., 2018).

### Other aspects

Moreover, BV and bvPLA_2_ had therapeutic and ameliorative effects on asthma, radial bone defect, dry eye disease and abortion, as well as eliminated teratogenic human induced pluripotent stem cells (iPSCs), as shown in Table 7.

BV prevented forkhead box A2 (FOXA2)-regulating mucin 5AC (MUC5AC) expression in IL-13-treated A549 cells not only through inhibiting Akt activation but also by suppressing enhanced SAM-pointed domain containing Ets-like factor (SPDEF), which demonstrated that BV could help prevent mucus metaplasia in asthma (Kim et al., 2021d). Although iPSCs is considerable practical in potential cell-based therapy, they remain confronted with problems such as teratoma formation. BV treatment resulted in impaired F-actin filaments within iPSCs, Ca^2+^ inward flow and accumulation to the cytoplasm, elevated calpain activity and reactive oxygen species (ROS) generation, selective induction and reduction of viability and adhesion capacity of undifferentiated stem cell iPSCs, and increased cell death. In addition, BV treatment before in ovo transplantation suppressed the formation of iPSC-derived teratoma. Moreover, BV was neither cytotoxic nor genotoxic to iPSC-derived differentiated cells. The above findings indicated that BV possessed anti-teratoma effect by getting rid of residual iPSCs, and could be a safe and effective cell preparation agent for stem cell therapy (Kim et al., 2020a).

BV alongside chitosan scaffold (CS) displayed high radiographic outcomes compared with the untreated and CS groups at day 56 postoperatively, and showed high osseous tissue density and high counts of osteocytes and osteoblasts compared with the CS and autograft groups, which highlighted the capability of CS-BV as a potential alternative and enhancer of bone graft substitute material (Meimandi-Parizi et al., 2018). The natural extract containing BV, deer antlers and musk as eye drops had therapeutic effects on scopolamine-injected dry eye disease rat models and lacrimal gland-excised dry eye disease rat models through repairing the damaged ocular surface, increasing the amount of tears, and restoring the tear mucin layer (Choi et al., 2020).

bvPLA_2_ prevented fetal loss with growth restriction in the remaining live fetuses in abortion mouse model induced by LPS. It increased Treg cells, and decreased TNF-α and IFN-γ expression. The prophylactic effect of bvPLA_2_ was not found in abortion prone mice lacking Treg. The above results indicated that bvPLA_2_ might have effects on LPS-induced abortion mice through regulating Treg populations (Baek et al., 2019).

## Safety and venom immunotherapy

An important concern with BV has been its safety. BV can cause anaphylaxis (Cherniack and Govorushko, 2018). It is reported that up to 3.4% of children and up to 7.5% of adults have systemic allergic sting reactions. Allergic reactions can be mild in skin; however, it can also be moderate to severe with a risk of life-threatening allergic reactions (Sturm et al., 2018). In an individual patient education, it addressed to correctly and timely use epinephrine auto-injectors, and recommend to stop physical exercise and adopt supine position during acute allergic attack (Stoevesandt et al., 2020).

Venom immunotherapy (VIT) could prevent severe allergic reactions in 80–100% subjects allergic to Hymenoptera venom. An immunological study on the early phase of VIT performed on forty individuals indicated that cytokines (CCL5/RANTES and IL-17E/IL-25) and eosinophils were conducive to the immune response (Palgan et al., 2020).

MEL could be used in adjuvant immunotherapy. MEL combined with LPS produced an increase of IL-1β and IL-6 levels and a decrease of IL-10 level in phorbol 12-myristate 13-acetate (PMA)-differentiated THP-1 cells. Besides, the response to MEL and LPS was characterized by metabolic profiling, and the clearest effects were on glycolysis, tricarboxylic acid cycle, oxidative phosphorylation, and purine, pyrimidine, and fatty acid metabolism (Alqarni et al., 2018). Similarly, the same research group also studied the metabolomic profiling for the immune stimulatory effect of a major constituent of BV, (Z)-11-eicosenol, and its derivations methyl cis-11-eicosenoate and cis-11-eicosenoic acid on PMA-differentiated THP-1 cells. The results supported the proposed actions of the three components as immune system stimulators (Alqarni et al., 2019). Besides, bvPLA_2_ has attenuated several immune system-related diseases. In a recent study, bvPLA_2_ decreased apoptotic Treg cells, reduced early apoptosis in splenocytes and CD4^+^CD25^+^ Treg cells, and raised the expression of CTLA-4 and PD-1 on CD4^+^CD25^+^ Treg cells, which showed that bvPLA_2_ could induce Treg expansion by changing apoptotic signal (Baek et al., 2020).

As experts have commented, if the venom is selected correctly, and adequate venom preparation and maintenance dose are used, VIT may be a treatment method of significant value in preventing the systemic reaction of Hymenoptera stings. Besides, the duration of VIT is usually 5 years, which can provide long-term protection from stinging after treatment interruption, but it recommends indefinite (possibly life-long) treatment for patients with mastocytosis or other high-risk patients (Incorvaia et al., 2018).

## Toxicity, administration dose and new drug delivery system

Although BV and its main components often show good results towards cancerous cells, bacteria, viruses as well as other disorders and diseases, there are always open questions regarding their potential toxicity on normal non-target or host cells and tissues making this kind of toxicity one of the biggest obstacles for the possibility of applying such natural products as medications. A single high dose of 100 μg/ml of BV damaged the DNA molecule in human lymphocytes *in vitro*, resulting in cellular instability (Garaj-Vrhovac and Gajski, 2009). A further study suggested that the DNA damage effects of BV could be at least partially related to oxidative stress (Gajski et al., 2012). In addition, BV decreased cell viability, altered cell morphology, as well as induced cytogenotoxicity and dominantly necrotic type of cell death, in human peripheral blood lymphocytes with a dose- and time-dependent manner (Gajski and Garaj-Vrhovac, 2011). Similarly, MEL was also found to induce cytogenetic damage and oxidative stress in human peripheral blood lymphocytes (Gajski et al., 2016).

Therefore, the administration dose is of great noteworthy. Wi-38, a species of human lung fibroblast, was less sensitive to MEL and has higher colony formation capacity than ChaGo-K1 cells (IC_50_ = 0.6 μM), with an IC_50_ value of 2.35 μM at 48 h (Tipgomut et al., 2018). After 72 h incubation, the cell viability of BESA-2B (normal lung epithelial cells) remained unaffected at MEL concentrations ≤4 μg/ml with some reduction in activity, while the viability of NCI-H441 and A549 cells (lung cancer cells) was significantly affected at 2 μg/ml (Gao et al., 2018). Besides, the Selective Index of NIH3T3 (healthy mouse fibroblast cells) was higher than 3 compared to HepG2 and MDA-MB-231 cells, implying that BV was more selective for both types of cancer cells and that NIH3T3 showed altered cell morphology only when BV was 100 μg/ml (Uzuner et al., 2021). Moreover, Melectin at around 15 μM only slightly inhibited NIH3T3; Concentration below 50 μM caused only 20% erythrocyte lysis, with no significant hemolytic effect (Liang et al., 2021). Comparable results existed for MEL-AF treatment, where MEL-AF remained non-significantly cytotoxic at doses in the range of ≤10 μg/ml after 24 h of MEL-AF treatment (Sangboonruang et al., 2020). Besides, NRK-49F (rat renal interstitial fibroblast cells) cell viability with 48 h apamin treatment was reduced at 10 μg/ml of apamin. However, 0.1, 0.5, 1, 2, and 5 μg/ml did not affect NRK-49F cell viability (Gwon et al., 2021).

The effect of MEL on the viability of MCF 10A (human normal mammary epithelial cells), HDFa (human dermal fibroblasts), and MCF-12A (human mammary epithelial cells) cells was more delayed and attenuated, with an IC_50_ value of 22.17 μg/ml for HDFa cells at 24 h (Duffy et al., 2020).

When BV and MEL treated THLE-2 (normal human liver cell line) for 24 h, the IC_50_ values were 95.73 μg/ml in the BV group and 78.38 μg/ml in the MEL group, both of which were significantly higher than the IC_50_ values of treated HepG2 (1.4 and 2.8 μg/ml, respectively), which showed negligible effects of BV and MEL on hepatocytes (Mansour et al., 2021).

After treating HaCaT (normal human keratinocytes) with 0.1–10 μg/ml of BV and MEL for 24 and 72 h, the toxic effects possessed by BV were more pronounced, with IC_50_ values reaching 6.72 and 6.38 μg/ml for the two time periods, respectively; the IC_50_ values of MEL-treated HaCaT were greater than 10 μg/ml exceeding the maximum MEL concentration used in the experiment (Nikodijevic et al., 2021).

BV and Wasp venom were non-toxic to BV-2 murine microglial cells at concentrations of ≤12 μg/ml and ≤160 μg/ml for 24 h, respectively (Yun et al., 2021). And 1 μg/ml of apamin showed a 10% decrease in BV2 microglial cell proliferation for 12 h (Park et al., 2020). Cell viability of 0.1–30 µM of MEL on HT22 mouse hippocampal cells for 24 h was assessed and 3 µM was found to be the maximum safe concentration for further study (Cong Duc and Lee, 2021).

BV at 0.1 and 1 mg/kg was an effective concentration and had no side effects on rats (Lee et al., 2020a). Similarly, 0.5 and 1.23 mg/kg of BV were also taken as therapeutic dosages to rats (Zahran et al., 2021). Besides, i.p. administration of 1, 2, and 4 mg/kg of MEL leaded to no toxicity in mice. The LD_50_ of mice with i.p. injection of MEL was 7.4 mg/kg (PubChem Database. MEL, CID = 16129627) (Fan et al., 2020).

In order to decrease the toxic and side effects such as hemolysis of BV and MEL, and improve the characteristics of drug targets and release, besides of NPs mentioned above (Zhou et al., 2021), other new drug delivery system has also been developed and applied in clinics (Lyu et al., 2019). After the amino acid modification of MEL or the addition of specific functional groups, it can improve the ability of MEL to target recognition and prevent degradation of target cells while reducing its toxicity to normal cells (Giribaldi et al., 2021). For example, after replacing valine at position 8 and proline at position 14 in MEL with lysine, MEL-pep had stronger ability to resist cell proliferation and inhibit tumor growth, reverse the drug resistance of cells to 5-fluorouracil, inhibit Akt pathway and P-glycoprotein (P-gp) of cells related to the exclusion of foreign bodies (Ke et al., 2018). Besides, a pH-sensitive amide bond was introduced between the 2,3-dimethyl maleimide and lysine of MEL to modify MEL, which could reduce the hemolytic status of MEL and maintain its low activity in the neutral or microalkaline environment, and show a stronger tumor targeting effect in the acidic microenvironment (Luo et al., 2018). The above investigations provided a feasible platform for improving the targeting and safety applications of BV and its main components, such as MEL.

## Conclusion and perspectives

BV and its main constituents have multiple biological activities and applied to treat several diseases, such as cancer, neurological disorders, inflammatory diseases, pain, microbial diseases, liver, kidney, lung and muscle injury, etc. In this paper, we reviewed the recently published reports (2018–2021) on BV and its main constituents. These articles have indicated that BV and its main constituents exerted the above protections and were contained in a variety of signaling tranduction pathways (Figures 2–4).

BV played a vital role in cell proliferation, migration, invasion, inhibition of EMT and induction of apoptosis and autophagy by exerting multi-pathway and multi-target long-lasting effects on cells in lung cancer, breast cancer, cervical cancer, and many other cancers *in vitro* and *in vivo*. The mechanisms of action of BV were: upregulation of Caspase-3, Caspase-7, Caspase-9, Bax, p21, p27, p53, Rb, PTEN, and 15-lipoxygenase-1 expression, downregulation of MITF, PARP, Bcl-2, Bcl-xL, ERα, EGFR, VEGF, MMP-1, MMP-2, MMP-9, TNF-α, NF-κB, HIF-1α, Cyclin D1, Cyclin A, Cyclin B, and Rac1, etc. BV inhibited the mTOR pathway, PI3K/Akt and MAPK signaling pathway, and mitotic signaling pathway by reducing the expression of p-mTOR, Akt, p-Akt, p38, p-p38, JNK, p-JNK, ERK, p-ERK, p-PI3K, p-EGFR, and p-HER2, which in turn impaired cancer cell viability, reduced migration and invasion activities, and promoted cell death. BV activated the mitochondrial apoptotic pathway in cancer cells by increasing the expression of apoptosis signaling molecules such as Fas and Caspase-9. On the other hand, it inhibited EMT in cancer cells by decreasing the expression of vimentin, ZEB2, and Slug and elevating the expression of E-cadherin (Figure 2).

Except cancer, BV and its main components can treat a variety of other diseases, including neurological disorders, inflammatory diseases, liver, kidney, lung and muscle injury, etc., mainly via inhibiting the release of inflammatory cytokines and chemokines, regulating the redox balance in the body, modulating the expression of apoptosis related factors, and so on. The most common points were: first, BV and its main components reduced pro-inflammatory cytokines (TNF-α, IL-1β, IL-6, IFN-γ, IL-13, IL-2, IL-12, IL-17, etc.) and raiseed anti-inflammatory cytokines (IL-10 and IL-4); second, they improved antioxidant capacity by upregulating the levels of GSH, GPx, SOD, CAT, glutamate-cysteine ligase (GCL), GST, etc., and downregulating the levels of lipid peroxidation (LPO), MPO, MDA, protein carbonyl (PCO), ROS, etc.; third, they downregulated pro-apoptotic factors (Caspase-3, cleaved Caspase-3, cleaved PARP-1, Bax, p53, apoptosis-inducing factor (AIF), Calpain, Cyt C, etc.) and upregulated anti-apoptotic factors (Bcl-2 and Survivin); fourth, they reduced chemokines, such as CCL17, CCL22, thymic stromal lymphopoietin (TSLP), macrophage inflammatory protein-1α (MIP-1α), MIP-1β, MCP-1, growth related oncogene-α (GRO-α), and so on; finally, they often downregulated MAPK, TLR4/NF-κB, Akt/mTOR and JAK/STAT signaling pathways, and upregulated Nrf2/HO-1 signaling pathway thereby producing corresponding pharmacological effects on different pathophysiological environments. Besides, BV and its main components upregulated TrkB/CREB/BDNF signaling pathway and the expression of growth-associated factors (BDNF, NGF, neurofilament 200 kDa (NF200) and growth-associated protein 43 (GAP43)), as well as downregulated the expression of amyloidogenic proteins (APP, BACE1, Aβ_1–42_ and Aβ_1–40_) in treating neurological disorders. They inhibited hepatic and renal fibrosis via suppressing TGF-β/Smad2/3 signaling pathway. They also downregulated the expression of C/EBPs and PPARγ, and activated AMPK signaling pathway in the treatment of obesity.

**FIGURE 5:**
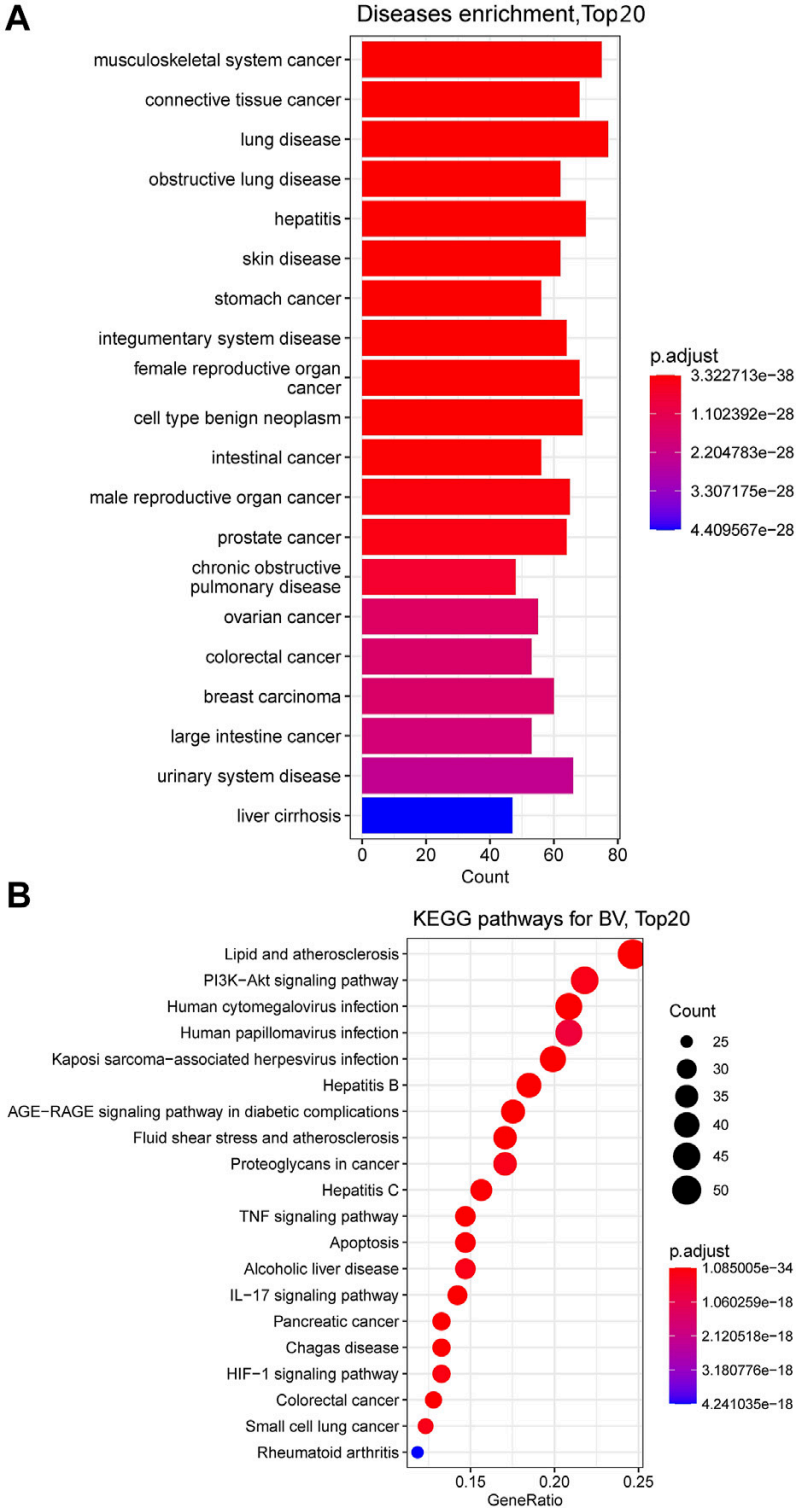
DOSE **(A)** and KEGG **(B)** analyses of proteins reported for BV and its main components in the literature. Top 20 diseases and KEGG pathways are enriched using R package “DOSE” and “topGO”, respectively with *p*-value Cutoff = 0.01, q-value Cutoff = 0.01).

**FIGURE 6:**
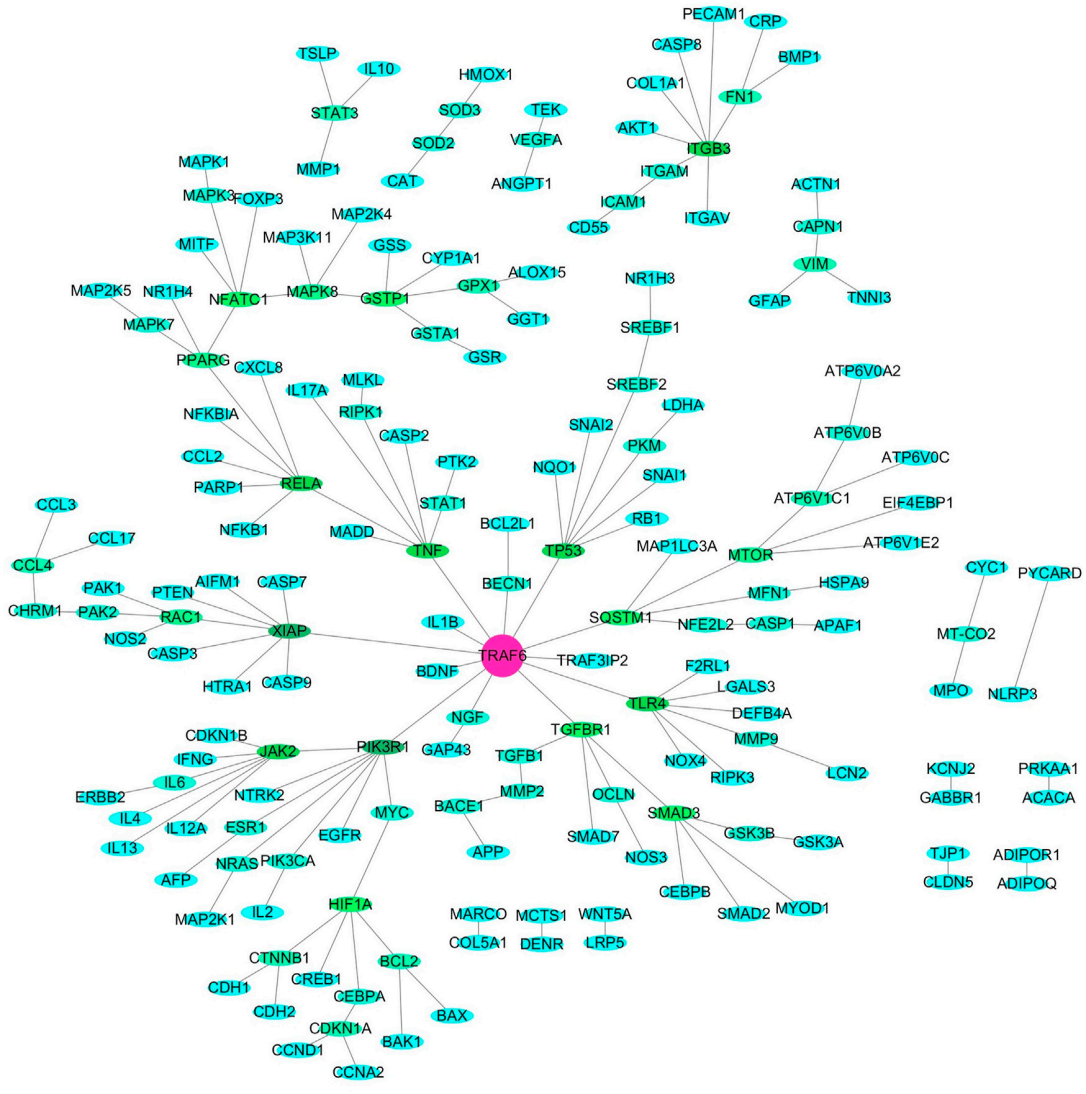
PPI analyses (confidence:0.4) of proteins and genes reported for BV and its main components in the literature visualized by Cytoscape 3.6.0 software. Texts in purple circle, green circle and light blue circle demonstrates the central hub target, secondary node target and the others node target.

The potential effects of BV and its main components on ills and their potential mechanisms were predicted through bioinformatic analysis, referring to DOSE analysis and KEGG pathway enrichment of the targets reported using bioinformatics tools in the light of our previous methods (Zhong et al., 2021; Lin et al., 2022). As shown in Figure 5A **,** beside the reported diseases by literature, BV and its main components maybe have effect on the connective tissue cancer and obstructive lung disease. Meanwhile, KEGG pathway enrichment analysis (Figure 5B) shows that BV and its main components can mainly regulate the proteins involved in the pathways of lipid and atherosclerosis, PI3K−Akt, Human cytomegalovirus infection, Human papillomavirus infection, etc. To further screen the pivotal targets, PPI analysis was performed with the reported proteins for BV and its main components using the STRING on-line sever (https://www.string-db.org). As shown in Figure 6, a central protein, TRAF6 is highlighted in the PPI “tree” (located in purplish red circle). Indeed, according to the KEGG database, this protein is truly involved in the regulation of up to 34 pathways, including MAPK, NF-κB, Pathways in cancer, etc. Moreover, seven subcentral proteins, X-linked inhibitor of apoptosis protein (XIAP), TNF, phosphoinositide-3-kinase regulatory subunit1 (PIK3R1), tumor protein p53 (TP53), SQSTM1 (p62), TLR4 and TGF-β receptor 1 (TGFBR1), which can be attributed to different functional pathways, are displayed the direct interaction with the central protein TRAF6.

Ultimately, based on the previous results and the present bioinformatics and PPI analysis, it gives the following perspectives.

(1) First, although all the reported targets were proved to be correlated with the interfere effects of BV and its main components mainly on cancers, the DOSE analysis strongly suggest that BV and its main components could have an effect on lung disease and obstructive pulmonary disease, which could be worth exploring in the current COVID-19 epidemic. Therefore, next, it is suggested to further validate the effect of BV and its main components on lung disease and obstructive pulmonary disease for seeking hope for the treatment of lung diseases, especially COVID-19.
(2) Second, the present KEGG enrichment analysis suggests that lipid and atherosclerosis, Human cytomegalovirus infection and Human papillomavirus infection pathways could be involved in the disease intervention, but these pathways are not mentioned in the previous reports for BV and its main components. For integratively understanding the intervention and mechanisms of BV and its main components on diseases, proteomics or genomics investigations should be performed using *in vitro* and *in vivo* disease models to confirm the unreported pathways in the future study.
(3) Last, TRAF6 is predicted as one core target and XIAP, TNF, PIK3R1, TP53, SQSTM1, TLR4 and TGFBR1 are predicted as subcentral proteins for the therapeutic effects of BV and its main components. Accordingly, it will be very meaningful to validate the target roles of these proteins for the further drug development of BV and its main components by gene silencing, knockout experiments, etc.

In conclusion, these ideas provide valuable clues or perspectives to further study on the pharmacological effects and mechanisms of BV and its main components, so as to the promoting the drug development and clinical applications for BV.
